# New policies on skin-to-skin contact warrant an oxytocin-based perspective on perinatal health care

**DOI:** 10.3389/fpsyg.2024.1385320

**Published:** 2024-07-09

**Authors:** Nils J. Bergman

**Affiliations:** Department of Women’s and Children’s Health, Karolinska Institutet, Stockholm, Sweden

**Keywords:** Kangaroo Mother Care, nurturescience, preterm birth, resilience, separation, vulnerability

## Abstract

**Background:**

In 2023, the World Health Organization (WHO) published a Global Position Paper on Kangaroo Mother Care (KMC), which is applicable to all countries worldwide: from the moment of birth, every “small and sick” newborn should remain with mother in immediate and continuous skin-to-skin contact (SSC), receiving all required clinical care in that place. This was prompted by the startling results of a randomized controlled trial published in 2021: in which 1,609 infants receiving immediate SSC were compared with 1,602 controls that were separated from their mothers but otherwise received identical conventional state-of-the-art care. The intervention infants showed a 25% reduction in mortality after 28 days.

**New perspectives:**

The new WHO guidelines are a significant change from earlier guidance and common clinical practice. The author presents that separating mothers and babies is assumed to be “normal” (a paradigm) but actually puts newborns at increased risk for morbidity and mortality. The author presents arguments and ethical perspectives for a new perspective on what is “normal,” keeping newborns with their mothers is the infant’s physiological expectation and critical requirement for healthy development. The author reviews the scientific rationale for changing the paradigm, based on synchronous interactions of oxytocin on both mother and infant. This follows a critique of the new policies that highlights the role of immediate SSC.

**Actionable recommendations:**

This critique strengthens the case for implementing the WHO guidelines on KMC for small and sick babies. System changes will be necessary in both obstetric and neonatal settings to ensure seamless perinatal care. Based on the role of oxytocin, the author identifies that many current routine care practices may actually contribute to stress and increased vulnerability to the newborn. WHO has actionable recommendations about family involvement and presence in newborn intensive care units.

**Discussion:**

The concepts of resilience and vulnerability have specific definitions well known in perinatal care: the key outcome of care should be resilience rather than merely the absence of vulnerability. Newborns in all settings and contexts need us to re-evaluate our paradigms and adopt and implement the new WHO guidelines on KMC in perinatal care.

## 1 Introduction

### 1.1 Revised WHO policy and guidelines on preterm care

New policies on Kangaroo Mother Care (KMC) and care of small and sick babies have been introduced by the World Health Organization in a Global Position Paper (WHO, 2023a) and an Implementation Strategy document (WHO, 2023b). This follows two recommendations presented in 2022, first that KMC “is recommended as routine care for all preterm and low birth weight infants” and should be given for as many hours as possible. Second, KMC “should be started as soon as possible after birth. *New, strong recommendation, high-certainty evidence*” (WHO, 2022b). The full set of recommendations are detailed in “WHO recommendations for care of the preterm or low-birth-weight infant” (WHO, 2022b). Evidence and recommendations begin with 16 recommendations under preventive and promotive care, and continuous Kangaroo Mother Care is the first recommendation with immediate KMC and the second one is followed by mother’s own milk and later a strong recommendation on “early initiation of enteral feeding.” Care for complications has six recommendations, and a final section is entirely new with four recommendations (two of which strong) devoted to “Family involvement and support.” An accompanying press release states that “This marks a significant change from earlier guidance and common clinical practice, reflecting the immense health benefits of ensuring caregivers and their preterm babies can stay close, without being separated, after birth” (WHO, 2022a). This article will present perspectives on preterm babies staying close with non-separation.

### 1.2 Background: brief history with notes on terminology

The role of skin-to-skin contact (SSC) in current perinatal care has evolved slowly since its introduction in 1979 in Colombia as the “Kangaroo Mother Method” by Rey and Martinez (1981). Their context was a severely overcrowded NICU with frequent nosocomial infections resulting in high neonatal mortality. As soon as the neonate was deemed “stable enough to tolerate skin-to-skin contact,” they provided that as long as possible, and then, they discharged the babies for continuous SSC with nutritional and breastfeeding support and supplementary oxygen if needed (Rey and Martinez, 1983). This required daily or frequent return visits to an outpatient support unit. The discharged infants had markedly better survival. Subsequently, Charpak led a team that elaborated this model into a center of excellence in Colombia (Charpak et al., 1994; Calume and Charpak, 1996).

The Kangaroo Mother Method was first described in English by Whitelaw and Sleath (1985). They pointed out that the survival was from a smaller selected group of survivors and not statistically correct. They titled their report as “the myth of the marsupial mother,” commending it to low-resource settings but having “nothing to teach developed countries about improving survival.”

Following the first publications from Colombia, Anderson and nursing colleagues visited and reported back to the USA (Anderson et al., 1986). The term Kangaroo Care (KC) established itself in the USA, evolving without a formal definition. It retained the concept that the baby had to be able to “tolerate” SSC but shed the breastfeeding support and early discharge components. A typical definition states “practice kangaroo care as soon as infants have achieved medical stability and are able to tolerate the transfer from the incubator” and recommends that “kangaroo care take place at least once per day for 1–3 h” (Stanford, 2024).

Anderson was first author of a Cochrane systematic review (Anderson et al., 2003) which was later updated (Moore et al., 2016) and examined the effect of early SSC on “healthy newborns” (inferred not preterm). Since term infants could “tolerate” SSC, starting in the first hour was possible. The main finding was improved breastfeeding. This strengthened the evidence base for the Baby Friendly Hospital Initiative in support of breastfeeding. The legacy (or paradigm) from Bogota and KC remained in that SSC was only provided for 1 h, after which necessary routines and cultural expectations restored maternal–infant separation.

Wahlberg and other nursing colleagues from Europe also visited Colombia (Wahlberg, 1986). They used the terminology of “skin-to-skin contact,” since this term was already in use and being researched in Sweden in the decade prior to Dr. Rey’s publication (De Chateau, 1976). Such research was ongoing and, in Sweden, led to earlier initiation of SSC for smaller babies in the clinical context and being provided for longer periods of time. This development may have been largely empirical in terms of perceived better outcomes and parental expectations rather than from evidence-based trials.

The term Kangaroo Mother Care (KMC) was originally conceived in 1996 at a WHO-sponsored meeting in Trieste, Italy (Cattaneo and Tamburlini, 1997; Cattaneo et al., 1998b). It was described as a care strategy for preterm and low-birthweight babies with three key components: Kangaroo Position, Kangaroo Nutrition, and Kangaroo Discharge (Cattaneo et al., 1998a). A KMC practice guide based on limited available evidence with early and diverse experiences was published in 2003 (WHO, 2003). This prescribes that for continuous SSC, the “baby’s condition must be stable.”

A first Cochrane review on KMC (Conde-Agudelo et al., 2000) defined according to the three components found improved breastfeeding and other outcomes but none for improved survival. Clinicians would be reassured about other outcomes which would matter little if neonates died from not tolerating the intervention early on. However, over time, studies were conducted and included in the review, in which SSC was initiated sooner after birth and for longer periods of time (Conde-Agudelo and Díaz-Rossello, 2016). Both earlier initiation and more continuous provision of SSC provided to low-birthweight infants once clinically stable (approximately 3 to 10 days) appeared to contribute to a 40% reduction in mortality.

### 1.3 The immediate Kangaroo Mother Care study (iKMC)

While there has been a widespread and growing acceptance, the actual implementation and uptake of KMC remains very low (Engmann et al., 2013; Medhanyie et al., 2019). This contributed to the WHO partnering with research funders to conduct three major studies. The results of these three studies (Mazumder et al., 2019; Arya et al., 2021; Mony et al., 2021) contributed significantly to the new guidelines from the World Health Organization (WHO, 2022b).

Given the earlier evidence for lowering mortality in stable babies and the very poor global uptake (Engmann et al., 2013), the first was an implementation study that sought to establish whether scale up to 80% coverage for all preterms was possible. Using mixed-methods application of implementation science with formative research, eight sites in India and Ethiopia succeeded in achieving the target (Mony et al., 2021).

Second, since many neonatal deaths occur outside of hospitals, a randomized controlled trial in Bangladesh looked at whether KMC could be initiated in the community. Babies between 1,500 g and 2,250 g were randomized to KMC or conventional care at a mean age of 30 h, and they received approximately 10 h of SSC per day. This resulted in a 30% reduction of mortality at 1 month (n 8,402; hazard ratio 0.70; 95% CI 0.51–0.96; *p* = 0,017), with survival benefit sustained to 6 months (Mazumder et al., 2019).

Third, the immediate KMC study (iKMC) was motivated by the fact that the great majority of preterm deaths occur before babies become clinically stable and eligible for KMC (Adejuyigbe et al., 2020). The study was conducted in Ghana, India, Malawi, Nigeria, and Tanzania. All hospital births were pre-screened for possible low birth weight, and pre-consent taken if this was likely, if infant was then born between 1,000 g and 1,799 g, consent was completed. Those randomized to intervention were started in SSC as soon as circumstances permitted. Controls continued receiving conventional care in delivery area or neonatal unit. The primary outcome was mortality, which was measured at 72 h and at 28 days.

#### 1.3.1 Further detail on conduct of iKMC study

Before the launch of the study, all five study sites received new and identical CPAP machines and monitors. All sites received intensive refresher courses in the use of these and in all the evidence-based aspects of care for preterm and low-birthweight babies compiled by the WHO (WHO, 2015; Kamath-Rayne et al., 2018). All sites received additional training in early and frequent expression and giving of colostrum (Parker et al., 2015), with early and ongoing support for suckling and breastfeeding.

Enrollment specifically included unstable newborns from birth, which guidelines at that time excluded from receiving KMC. The intervention group received the exact same package of care, but with extra training provided for use of technology and care while on the chest of mother or surrogate in SSC, provided in an adult hospital bed in the NICU from the first hour after birth. Necessarily and unavoidably, training and support in caring for the baby was also provided to the mothers and surrogates chosen by her. This entailed bringing not only mothers but other family members into the NICU. Continuous SSC also required that mothers or surrogates slept while providing SSC, with safe technique ensured by the use of a “KMC garment.” This consisted of a binder of non-stretch cotton material that ensured the infant’s patent airway and a wraparound shirt ensuring fixation in a fetal position on the mother’s chest. To implement the SSC intervention, teams of “KMC supporters” were recruited with research funding. All sites were large and busy tertiary centers with high patient-to-nurse ratios. Not one of the parts of the intervention was in current job descriptions, and it was not possible to add any tasks to nurse workload. KMC supporters were present at enrolment and supported clinical staff in placing baby in SSC having been trained in the use of the KMC garment. They coordinated continuous SSC with surrogates and practical support for extended stay of the family in the NICU. They were also tasked with very early and frequent regular colostrum expression and taking this to the infants. Such colostrum collection could precede enrollment and initiation of SSC and was done the same to both groups of mothers. This specifically ensured also that breastfeeding was controlled in the study design.

An objective definition of clinical stability based on a composite of clinical parameters was collected 6 hourly and used consistently. Intermittent KMC in short periods was given before stability (Nyqvist et al., 2010). Once clinically stable for 24 h, infants in both groups were moved to a common KMC ward. For stable small and sick newborns, an evidence-based reduction in mortality from SSC already existed (Conde-Agudelo and Díaz-Rossello, 2016). Neonatal consultant visits took place quarterly throughout the study to ensure evidence-based guidelines were followed and provided without differential to both groups. Essentially, both groups received optimal evidence-based medicine according to the state-of-the-art standards.

#### 1.3.2 Results of the iKMC study

**Figure 1:**
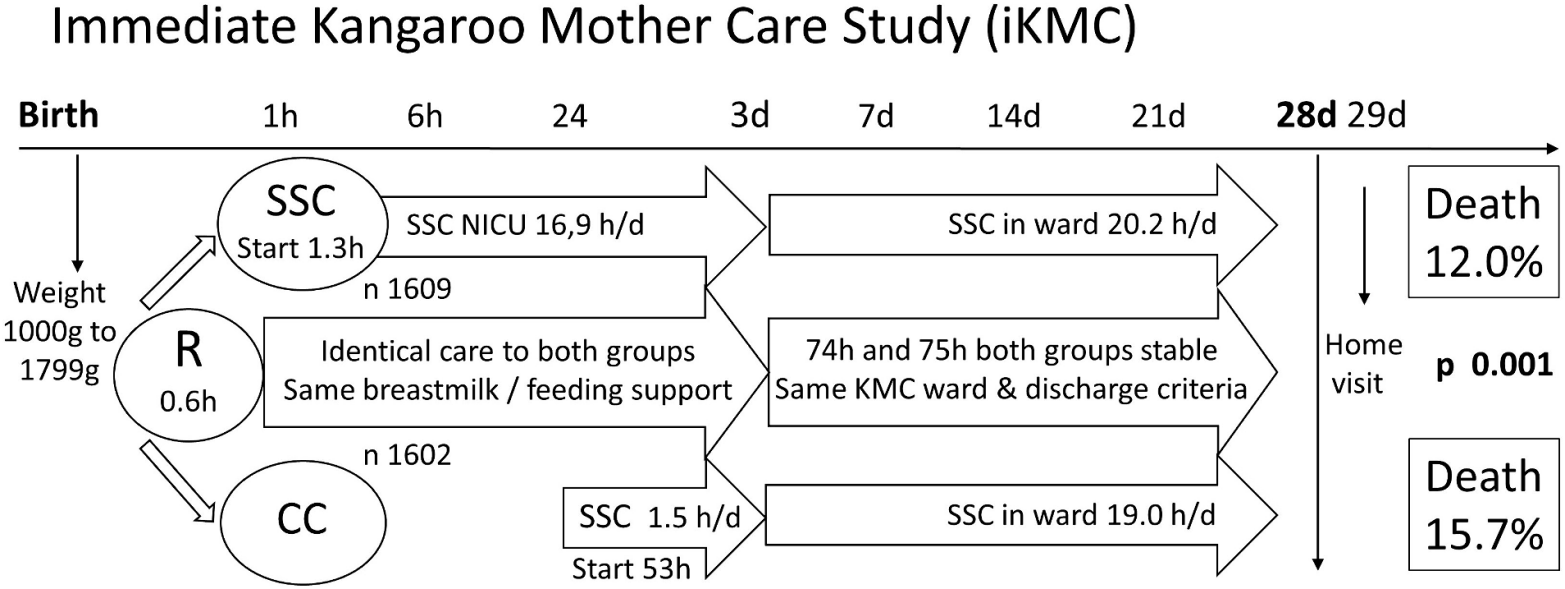
Immediate Kangaroo Mother Care Study (iKMC). The iKMC study required birthweight prior to randomization (R), one arm to skin-to-skin contact (SSC) and the other to Conventional Care (CC). There was no difference in time to reach clinical stability. Neonatal mortality outcome was captured on day 29 of life. Values are means only, h hours, h/d hours per day, n number.

Enrollment to the study was halted at 75% of recruitment target (3,211 instead of 4,200), after interim analysis by the data and safety monitoring board showed “clear benefit in neonatal survival in the infants receiving immediate kangaroo mother care” (Arya et al., 2021). The control group in this study started SSC earlier than in any studies on which this evidence is based, (mean 53 h, even before clinically stable at 75 h mean age), and they got it for longer daily doses thereafter. The study is presented graphically in Figure 1.

The result was a 25% reduction in mortality (n 3,211; risk ratio 0.75; CI 0.64–0.89; *p* = 0.001) from immediate KMC compared with traditional KMC (Arya et al., 2021). Less hypothermia contributed to this (already evidence-based), but the difference was primarily driven by a decrease in sepsis and a better survival in the presence of sepsis. All other secondary outcomes including exclusive breastfeeding rate at 28 days were the same in both groups, reflecting the study design intention to control for care and feeding in both groups.

## 2 New perspectives on KMC

### 2.1 Perspective in terms of separation

The iKMC study contributed to the new WHO policies, and as quoted above “reflecting the immense health benefits of ensuring caregivers and their preterm babies can stay close, without being separated, after birth” (WHO, 2022a). A new perspective can be discerned from this. “Without being separated” directly challenges the current concept of normality that separating small and sick newborns from their mothers is normal and necessary. The iKMC study was not designed as a separation study, but the two arms are essentially mother–infant togetherness compared with mother–infant separation. The conceptual novelty is to flip the understanding of normality (the control part of the RCT) as being skin-to-skin contact, and that of separation being the intervention to be studied. In statistical terms, the iKMC result would then be reported as separation resulting in a 33% increase in mortality (from 12 to 16%).

In mammalian and primate research, maternal–infant separation is used to study stress, being the most severe stressor known to infants. The documented harmful effects of social deprivation in non-human primates are global, affecting all neurobiological systems (Kalin and Carnes, 1984; Parker and Maestripieri, 2011), with pathology that “persists into adulthood and cannot be cured” (European Commission, 2002).

### 2.2 Perspective in terms of paradigm

The separation perspective can be identified as a paradigm. This has been defined as “an entire constellation of beliefs, values, and techniques, and so on, which is shared by the members of a given community” (Kuhn, 2008), further a “set of assumptions, concepts, values, and practices that constitutes a way of viewing reality for the community that shares them, especially in an intellectual discipline” in the American Heritage Dictionary. In terms of a “way of viewing reality,” health practices of the obstetric and neonatal community in the last 100 years have been based on the assumption and belief that maternal–infant separation is normal. This separation is entrenched in the technological environment of the NICU with strict hygiene control. This “separation paradigm” means that although research shows that SSC has benefits, it is an intervention that is not part of “normal” care and therefore does not fundamentally challenge the status quo. The results of the iKMC study are a challenge to the status quo, since separation is evidently contributing to an increase in neonatal mortality, the opposite of professed goals.

The disciplines of obstetrics and neonatology observe themselves firmly rooted in scientific rigor and evidence-based medicine. Nevertheless, there is a maternal–infant separation paradigm underlying current perinatal care, which is an assumption and an unquestioned way of viewing reality. This article will argue that this paradigm lacks both scientific rationale and evidence base.

In the past, neonatal outcomes were deemed to be good based on improving survival, assuming that brain development took place later. Current care has shifted to looking at the quality of neurodevelopmental outcomes (White, 2004), and it is increasingly clear that outcomes are poor and not improving (Twilhaar et al., 2018; Pierrat et al., 2021; Louis et al., 2022). In terms of “reflecting the immense health benefits” (WHO, 2022a), almost all articles on SSC research report “benefit” from SSC. The perspective presented here is that when SSC is regarded as the “normal,” separation outcomes would be reported as “harm,” with adverse effects on the global physiology and psychology of the neonate and the mother. Current health care is focused on decreasing risk and harm, evidence-based medicine being presented as “risk reduction.” The benefit aspect may appear less important or even not considered.

Paradigms are powerful, and they can change. Ignaz Semmelweis presented evidence that handwashing decreased mortality from puerperal sepsis. He could however not provide any acceptable scientific rationale (plausibility) for his findings, so he and his findings were rejected. Only after his death was the “germ theory” discovered, handwashing became accepted (Bergman, 2019b). Wikipedia defines the “Semmelweis reflex” as the “reflex-like tendency to reject new evidence or new knowledge because it contradicts established norms, beliefs, or paradigms.” These new recommendations with an evidence base are in direct contradiction to the maternal–infant separation paradigm, which is recognized by the statement that “this marks a significant change from earlier guidance and common clinical practice” (WHO, 2022a). In view of the Semmelweis reflex operating, the new evidence and recommendations may not be enough to make practice change. A new paradigm or way of thinking is needed, and unlike the time of Semmelweis, this new evidence does indeed have an acceptable scientific rationale.

### 2.3 Perspective in terms of SSC as the “place of care”

#### 2.3.1 Underlying science of place

Panksepp summarizes the “central dogma” of all psychobiological processes: the DNA is transcribed to RNA, which translates to proteins and the development of the brain and the behaving body: everything else follows, and the environment “permeates all phases of these transactions” (Panksepp, 1998). Repeating this in other terms, the epigenes read the environment to adapt gene expression to that specific environment. The sensory environment stimulates brain pathways to fire and consolidate in a final connectome, and then, the brain directs the body in behaviors best suited for that same environment (Bergman et al., 2019).

Instincts have not always been accepted as important for clinical practitioners in our current paradigm. Instincts are “highly conserved neuroendocrine behaviors.” The highly conserved refers to the deep and ancient code of the DNA (hardware), the neuroendocrine covers neurotransmitters in the brain and hormones in the body expressing behaviors (software), the resulting behaviors ensure that the organism is safe and will thrive in that environment. Reflexes are more accepted as they can be observed. Reflexes can be elicited by appropriate stimuli, and the integrity of neurological pathways can be tested. However, reflexes can be elicited in the absence of salience or relevance to context and confuse the organism. Salient stimuli elicit fundamental behaviors more than reflexes, for well-being and development first and foremost (e.g., first hour prefeeding behavior and suckling at breast) and second for survival (e.g., vigilance, freeze, and dissociation). The expected salience and context for human infant behavior is the mother’s body. The full epigenetic and sensory input for expressing developmental behavior is provided by direct SSC, and this behavior is first evident in suckling as a step toward breastfeeding.

#### 2.3.2 First immediate SSC experience

In 1988, the practice of KMC or SSC was described in only six publications. This author started working in a remote rural hospital in Zimbabwe without access to incubators for stabilizing low-birthweight infants. Each preterm infant was therefore placed immediately after birth on the mother’s chest, dried, and covered to keep warm with observations and indicated care (Bergman and Jurisoo, 1994). After the first hour or two, the infant was secured onto the mother’s chest with a KMC garment (see Section 2.3.1). After 1 h, the garment was loosened and infant was fed mother’s own milk and then placed back in the KMC garment. Other medical care was provided to the full extent possible in the low-income country rural setting. Over the next 5 years, 126 small babies were born in or admitted to the hospital. Compared with historical control records of good quality from the previous 4 years, very low birth weight infants (between 1,000 g and 1,499 g) had a 40% decrease in mortality (from 50 to 10%) (Bergman and Jurisoo, 1994). What was also apparent was a different demeanor of the infant: an alertness and personality presence with remarkable eye-to-eye contact.

#### 2.3.3 Further science on place

Historical control trials have no evidence base value; randomized controlled trials are necessary. In preparing to undertake such a trial, a literature review showed that there was a very extensive body of knowledge on maternal–infant separation, albeit only in mammals and, specifically, in non-human primates (Caine and Reite, 1981; Codner and Nadler, 1984; Kalin and Carnes, 1984). At that time not much was known about epigenetics and the genome, but neurodevelopment and subsequent behavior had been mapped in detail. Alberts asserts that learning and behavior “cannot be fully understood separately from a behaving body” (Alberts, 1994), and that development is characterized by a sequence of transitions of developmental habitats (e.g., for rats: uterus, mother’s body, nest, and littermates). “Developmental adaptations evolved in contexts that differ from our modern environments, (if) evolutionarily unexpected may inadvertently create pathology” (Alberts, 1994). Similar assertions came from Hofer, describing early relationships as regulators of infant physiology and behaviors (Hofer, 1994a,b) and maternal separation being stressful due to loss of such regulation, with adverse effects over the lifespan (Hofer, 2006). For contrasting healthy development, maternal–infant separation was universally used as a tool to show harmful changes to brain structure, pathways, and behavior. An example is as follows: twice daily, 3 min of separations for 3 days was enough to induce depression in a rodent model (*Octodon degus*) to test antidepressants for human use (Ziabreva et al., 2003).

In human studies, Porges described the role of the autonomic nervous system in emotion, the polyvagal theory, and its evolutionary roots (Porges, 1997). Perry reviewed childhood trauma and the neurobiology of adaptation (Perry et al., 1995), presenting a detailed analysis of the acute responses to stress. Perry introduces this article with the statement “adults interpret the actions, words, and expressions of children through the distorting filter of their own beliefs.” This latter phrase may summarize the separation paradigm of perinatal care: a distorting filter of beliefs.

Possibly the totally over-riding function of the brain is to ensure survival. This requires identifying threat and responding rapidly. It is variously described in the literature, often as “threat appraisal” (Gunnar and Quevedo, 2007); Porges introduces the term “neuroception” (Porges, 2003); perhaps the simplest is to ask “Am I safe?” The answer comes from an ongoing sub-cortical primary assessment of all available external and internal sensory inputs to the brain and their contribution to physiological regulation. This decision is based on perception and assessment and is totally true for that individual at that moment. It is not to be confused with the medical, clinical, and institutional definitions or paradigms of patient safety. The perception of threat immediately begins suspending the physiological processes of well-being and growth, not totally for distal threat but also increasingly and rapidly commensurate to threat proximity (Graeff, 1994; Perry et al., 1995). Perry describes an initial stage of vigilance for distal threat, if the threat is close a freeze response follows, if threat overwhelming then dissociation. Each stage is mediated by deeper limbic systems (Perry et al., 1995). Porges terms similar stages “safe, dangerous, or life-threatening” (Porges, 2003). In mammalian studies, equivalent terms used are protest and despair (Levine et al., 1985). Bowlby in human infants used the same terms with an addition: detachment (Bowlby, 1980; Hofer, 2006); this still in current use: “three theorized response phases—protest, despair, and detachment—that follow place attachment disruption” (Counted et al., 2021).

#### 2.3.4 The place model—SSC as the right place

**Figure 2:**
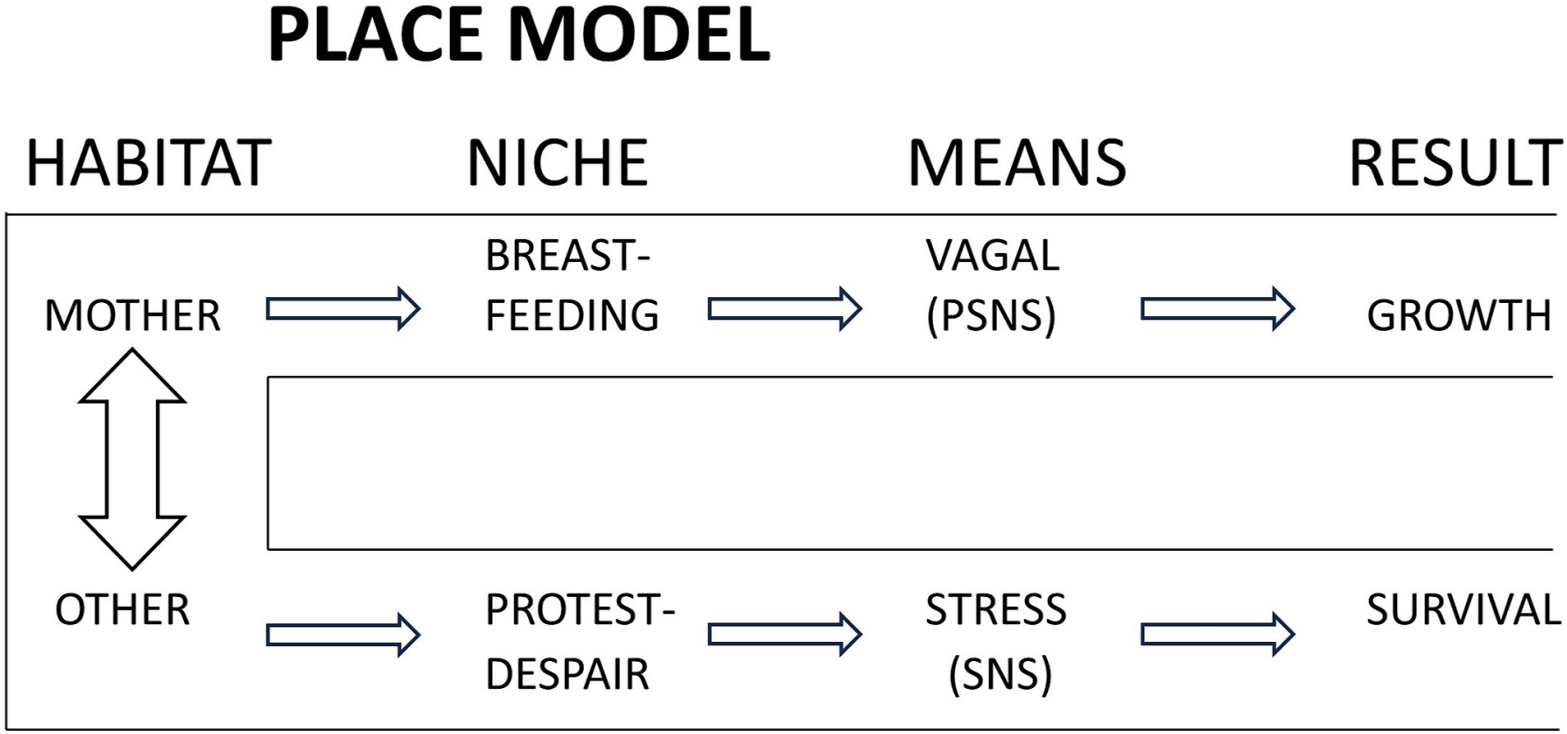
The place model. The expected place or habitat for the newborn is the mother, in which are expressed a set of behaviors, mediated primarily by the parasympathetic nervous system (PSNS) for optimal growth and development. Separation from mother, to any other place, results in stress mediated responses mediated by the sympathetic nervous system (SNS), augmented by PSNS activity (not shown). Reproduced from Bergman et al. (2004) with permission from Wiley.

In developing the above science to a testable research hypothesis, the place model was the result (Morgan et al., 2011) (see Figure 2). The terms “habitat” and “niche” came from Alberts (1994), denoting place and resulting behavior. The utility of the model is to emphasize the primary role of mother as place and then “viewing reality” in this paradigm to recognize separation behavior distinct from expected behavior. Furthermore, a study design can expressly control for all other care or circumstances, apart from the place, habitat, or environment.

Attributing the effect of parasympathetic nervous system (PSNS) only to being with mother and sympathetic nervous system (SNS) to separation is an oversimplification but as drawn here seeks to convey verifiable and measurable processes of the hypothesis. “Stress” covers the diverse levels of responses to threat described by Porges and Perry (Perry et al., 1995; Porges, 1997). The hypothesis formulated was that separation from mother would be perceived as threat and lead to responses identifiable as such. Maternal presence would first express as improved regulation and transition to extrauterine life, with early suckling and improved breastfeeding.

#### 2.3.5 First randomized controlled trial on immediate SSC

**Figure 3:**
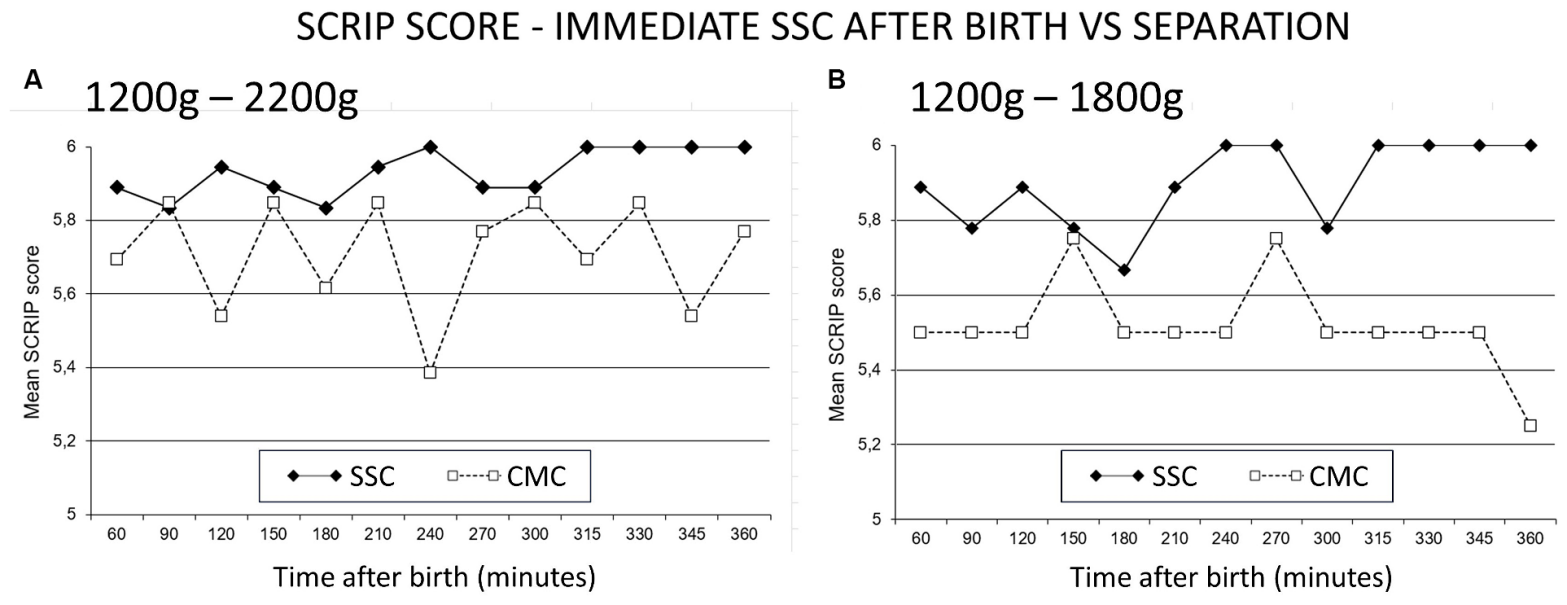
SCRIP score—immediate SSC after birth vs. separation. The Stability of CardioRespiratory System In Preterms (SCRIP) score assigns 0, 1 or 2 points for heart rate, respiration and oxygen saturation parameters, adding to 6 to be deemed stable. **(A)** Infants between 1,200 g and 2,200 g randomized to SSC (skin-to-skin contact) were stable at 6 h, those in CMC (conventional method of care) did not achieve the full score. **(B)** Infants 1,200 g to 1800 g in CMC were more unstable at 6 h. Reproduced from Bergman et al. (2004) with permission from Wiley.

This study expressly sought to control for care and circumstances, with only place being the variable studied. The separation paradigm requires infants to be “stable enough to tolerate SSC” (WHO, 2003). There was however no objective measure or definition in the literature that defined “stable.” The primary outcome of the RCT was therefore any one objectively identified clinical sign of marked instability (high specificity). In addition, a sensitive outcome measure for stability was designed based on a composite of cardiac and respiratory observations. A “Stability of CardioRespiratory System In Preterms” (SCRIP) score had been used for stable neonates (Fischer et al., 1998), and this was modified to cover observations for the first 6 h, guided by the clinical observation in Zimbabwe that this was the time required for transition to extrauterine life in SSC (Bergman and Jurisoo, 1994). Infants were recruited prior to expected preterm birth, and were kept in SSC without separation until weighing and confirmed to be between 1,200 g and 2,200 g and then randomized to continue in SSC or to conventional care in incubator (Bergman et al., 2004). Both groups spent the first hour in the same delivery ward, and the next five in the same neonatal ward, receiving the same standardized care. The following figures from the publication support the hypothesis (see Figure 3).

SSC ensures regulation with optimal scores at 6 h. Separated infants remain unstable, and the smaller they were, the more dysregulated they became. The study was replicated in 100 infants in Vietnam, confirming these findings with more details (Chi Luong et al., 2016). The novel perspective or paradigm is that the instability is directly due to being separated, and it is separation that a neonate does not tolerate. When moved from the incubator to mother, they might take time to settle, especially if moved supine in a bright and noisy environment. An infant in deeper dissociation or despair will appear unstable while moving through the dangerous or protest phase (Levine et al., 1985; Porges, 2003). The reaction reflects the high state of cortisol, which can take 30 to 60 min to wash out of system (Mooncey et al., 1997; Modi and Glover, 1998; Grunau et al., 2005).

#### 2.3.6 Physiological studies on place model

The place model was the rationale for more detailed research studies using heart rate variability (HRV), impedance cardiography, and EEG, and only the HRV is published (Morgan et al., 2011). In 16 2-day-old infants, awaiting discharge after caesarean section, acting as their own controls, place was alternated with SSC and with a crib next to mothers’ bed, with place order randomized. With control for level of state organization, separation showed profoundly increased autonomic nervous system activation, which was very similar to the mammalian protest and despair response (Levine et al., 1985). Furthermore, there was a profound reduction in sleep (86% less Quiet Sleep), which was attributable to anxious arousal (Morgan et al., 2011). Noteworthy in the above publication, and relevant for the paradigm presented in this article, is that the formulation of methods and results is based on the normal defining place being mother, and evidence of harm from separation in human infants is being studied and identified.

In summary, the place influences all aspects of genetic, cellular, neurological, and behavioral development (Panksepp, 1998). Threat appraisal is an overriding function of the organism. Perception of safety is place-dependent, and for human newborns, that place is mother’s chest. Separation leads to an immediate stress response.

### 2.4 Argument against maternal-neonate separation

A detailed argument for avoiding maternal-neonate separation has been published (Bergman, 2019a). Specifically, in the care of small and sick neonates, the direct consequence of separation is toxic stress (Sanders and Hall, 2018), which is defined as the “absence of the buffering protection of adult support” (Shonkoff and Garner, 2012). Stress can be positive: in small and brief doses, it allows for learning stress resistance (resilience), as long as maternal regulation is ongoing to provide healthy physiological setpoints (Hofer, 2006). However, stress must be buffered, and in the context of the neonate, the “adult” is more naturally the “mother” (newborn buffering can however be done by others). Tolerable stress can be moderate and even severe in intensity, but adult buffering allows for coping (Shonkoff and Garner, 2012). In the absence of buffering support, cortisol is released and works to ensure self-regulation, which is expressed as homeostasis (Hofer, 2006). These high cortisol levels achieve the “stable vital signs” measured in separated neonates (Mooncey et al., 1997).

Meaney and his team conducted iconic experiments with high grooming and low grooming rat dams influencing cortisol receptor expression in offspring (Weaver et al., 2004; Champagne and Meaney, 2006). When cortisol in earliest life remains high, the genes for the expression of cortisol receptors become increasingly switched off by epigenes (methylated), keeping that cortisol high over the lifespan (Meaney and Szyf, 2005). Early adversity “accelerates the development of amygdala-prefrontal cortex development and modifies emotional behaviors” (Callaghan et al., 2014). To the neonate, the reason mother does not provide more caregiving is not that she is a bad mother but a (good) signal that the outside world is harsh and difficult (bad), requiring adaptation to a harsh place or environment (Ellis and Del Giudice, 2019).

Epigenetic changes in human babies in association with caregiving have been described for cortisol in the same way but also for oxytocin receptors, immune T cell responses, and glucagon metabolism (Wigley et al., 2022). Early life experience profoundly influences oxytocin and receptor systems, showing reduced activity after early-life stressful stimuli (Onaka and Takayanagi, 2021). This has been dubbed as signaling (Kenkel, 2021), whereby maternal environment and behavior in the first hours of life become the messaging to the adapting neonate about the state of the outside world (Ellis and Del Giudice, 2019). This signaling can be followed by “canalization,” with changes being “very early, once off, and forever” (Morgan, 2013). Perry describes in detail how an early state of fear becomes entrenched as subsequent trait (Perry et al., 1995). Keeping high cortisol may be a “predictive adaptive response” that is helpful for survival (Gluckman and Hanson, 2005), particularly if the world turns out to be bad, as predicted. Such responses occur whenever a gene expresses during development, not only at birth but also the prediction may be wrong, in which case there is a maladaptation. The high cortisol intended to manage the outside environment instead wreaks havoc on the internal environment, the development, physiology, and health (McEwen and Seeman, 1999). Allostatic load may operate from very early also but has similar mechanisms of operation throughout the life span. The end result is vulnerability. This is the underlying rationale for the whole new discipline of Developmental Origins of Health and Disease (DOHaD) (Hochberg et al., 2011; Heindel et al., 2017). An unsafe environment makes stress management with cortisol the overriding priority of the organism, with survival at the cost of longevity with early and higher reproduction (Teicher et al., 2002).

### 2.5 The broader oxytocin-based perspective

A key role of cortisol (among many others) is managing threat in an unsafe environment. In contrast, as much as the “environment permeates all phases” of biological processes (Panksepp, 1998), oxytocin also permeates all phases of optimal development and reproductive biology. The critical importance of the safe environment, and the place model described above, is intricately and inseparably linked to the role of oxytocin. Optimal development and the capacity to thrive require a safe environment, and the infant’s highly conserved neuroendocrine behaviors are dependent on its own oxytocinergic system (Buckley et al., 2023).

Reviews on the broader aspects of oxytocin describe its origins and chemical features. Oxytocin has evolutionary origins going back 500 million years, being involved in water conservation, thermoregulation, and energy balance (Feldman, 2020). Over time, the oxytocin system expanded in “mammals, to manage over-reactivity to the ‘side effects’ of oxygen, including inflammation, oxidation, and free radicals, while also supporting high levels of sociality and a perception of safety” with close linkage to the autonomic nervous system (Carter and Kingsbury, 2022). Oxytocin is involved in pair bonding, possibly first appearing in some species of birds, which have larger brain-to-body size ratios than birds with other breeding patterns (Schultz and Dunbar, 2007). This is observed in other mammalian species, with the exception of later evolving primates, where brain size increases proportional to group size (Dunbar, 2009). The resulting “social brain hypothesis” is based on oxytocin (Norman et al., 2012). Subsequently, oxytocin took on a key role in labor and lactation (Feldman, 2020), being the platform for sociality as described above (Moberg et al., 2020). Carter summarizes additional functions: “resilience and healing, stress-coping, anti-inflammatory, and antioxidant, with protective effects especially in the face of adversity or trauma” (Carter and Kingsbury, 2022).

#### 2.5.1 Infant oxytocin

For the human newborn, the necessary environment is the mother and, specifically, maternal–infant skin-to-skin contact (Bergman, 2014). The role of the oxytocinergic system and related neuroendocrine mechanisms operating in SSC has recently been reviewed (Moberg et al., 2020). All maternal sensory inputs and pathways contribute to stimulating oxytocin in the newborn, activating a broad “calm and connection system.” Particular to SSC are the cutaneous sensory nerves activated by touch, light pressure, and warmth, and that, the latter has an element of pulsatility which contributes to regulation and a sense of trust and safety. SSC also directly stimulates mechanosensitive C-fibers (Moberg et al., 2020), which is identified as the substrate for “affective and rewarding properties of touch” (McGlone et al., 2014). This “decreases levels of fear and stress” and is also the prerequisite for development of social interaction. There follows an “enduring shift in the balance of the oxytocinergic system … and the stress system.” The parasympathetic system is directly and broadly activated to support the entire physiology, achieving “peripheral and central mechanisms related to restoration and growth” (Moberg et al., 2020).

#### 2.5.2 Adult oxytocin

Social science reviews focusing on adults present the role of oxytocin on sexual behavior, maternal, and caregiving toward newborns in mothers and others, with “subtle social processes … social memories formation, aggressiveness toward strangers, and anxiety reduction” (Ramalheira and Conde Moreno, 2022). Maternal aggression is specific to defense of young (Bosch et al., 2005). SSC has been shown to have long term effects on oxytocin in mothers and fathers in parent-infant interactions (Scatliffe et al., 2019). The serotonergic system interacts with oxytocin, impacting mood and parenting sensitivity (Bakermans-Kranenburg and van Ijzendoorn, 2008). Dopamine systems interact with oxytocin, and when this is disrupted, it may contribute to neuropsychiatric disorders (Baskerville and Douglas, 2010). The concept of the “evolved developmental niche” (Narvaez et al., 2016) or the “evolved nest” covers the broad neurobiological and sociomoral development of infants to adulthood (Tarsha and Narvaez, 2023). Oxytocin is identified as involved in all evolved nest domains, which is described as “soothing perinatal experiences, breastfeeding, positive touch, responsive care, multiple allomothers, self-directed play, social embeddedness, and nature immersion” (Tarsha and Narvaez, 2023).

#### 2.5.3 Maternal–infant synchrony

The infant oxytocinergic system is triggered by the mother, and the infant simultaneously triggers the maternal oxytocinergic system (Levy, 2016). Hofer describes interactions based on sensory exchanges that achieve physiological regulation of the infant (Hofer, 2006). These interactions are based on “physiological mechanisms, in particular oscillator systems” (Feldman, 2007). Twins in simultaneous skin-to-skin contact show differential thermal synchrony, one twin can be cooled by one breast while the other breast warms the other twin (Ludington-Hoe et al., 2006b). Oxytocin controls maternal temperature in a pulsatile manner, which is identified as an early experience of trust (Moberg et al., 2020). This reflects a psychobiological effect leading to “positive affective involvement … mutuality and reciprocity in the dyad” (Bystrova et al., 2009). The oxytocin pulsatility is identified as a key mechanism in the early sensitive period of birth, along with prior priming from pregnancy and “neural plasticity at the molecular and network assembly levels” (Feldman, 2015). SSC induces an enduring strengthening of the oxytocinergic system to counter the stress system (Moberg et al., 2020). This leads to mutual synchronous and reinforcing simultaneous oxytocin-related behaviors, each potentiating each other (Apter-Levi et al., 2014). Feldman defines synchrony as the “the coordination of biology and behavior during social contact” (Feldman, 2015). Social synchrony “is learned within the parent-infant bond” (Apter-Levi et al., 2014). This synchrony underlies the regulation, buffering, emotional connection, and resilience of the infant, as well as maternal neuroplasticity.

In summary, this broad spectrum of oxytocin effects shows that oxytocinergic systems interact with almost all other systems, at neurological and hormonal levels (Feldman, 2020). In particular, this applies to maternal and infant regulation and stress management (Charney, 2004; Feldman, 2020) and social synchrony (Apter-Levi et al., 2014).

### 2.6 Oxytocin-based perspective on immediate SSC

In the following sections, key behaviors or responses following birth in the absence of separation—in the “right place”—are presented. This is informed by the central dogma of biological sciences (Panksepp, 1998), in which the environment allows for expression of highly conserved neuroendocrine behaviors. Awareness of these allows recognition of them in observations of non-separated infants, and more insights will undoubtedly come to light in the future. Oxytocin is already identified as the common thread in each, and some additional aspects will be described. The order of each as described is approximately chronological, but all are interrelated.

That the immediate KMC study should have such an impact on improving survival in preterm infants was unexpected, given the separation paradigm identified. The key role of the biologically expected place and the resulting perception of safety have been described above, with the broad effects of oxytocin that follow. The review on neuroendocrine mechanisms and physiological effects caused by SSC mentioned above (Moberg et al., 2020) describes a host of beneficial effects. Those mechanisms have in our current health systems only had brief opportunity to function, particularly for small and sick babies provided traditional KMC or Kangaroo Care with brief episodic SSC. Since preterm infants have spent the greater part of their first hours and days separated, our understanding of their expected behavior with immediate and continuous SSC—without separation—is limited.

#### 2.6.1 Regulation

The evolutionary origin of oxytocin was focused on regulation, fluid, and energy balance with temperature regulation (Feldman, 2020), and this is the downstream or physiological regulation. The mammalian evolution coopted oxytocin for reproduction and sociality, an upstream regulation. Carter summarizes that oxytocin manages “over-reactivity to the ‘side effects’ of oxygen, including inflammation, oxidation, and free radicals, while also supporting high levels of sociality and a perception of safety” (Carter and Kingsbury, 2022). Furthermore, the oxytocin system interacts with other systems to manage threat, fear, and stress (Charney, 2004).

Stress hormone levels are extremely high at birth and vital for neonatal adaptation to extrauterine life through gene activation and activation of the locus coeruleus for being awake to bond to mother (Lagercrantz, 1996). They are also activating sodium channel pumps for clearance of the lungs (De Luca et al., 2009.). Oxytocin is also extremely high at birth and is needed to lower the high cortisol. Maintaining oxytocin requires ongoing buffering protection from mother. Hofer attributes this regulation by the whole sensory environment as “hidden maternal regulators,” and oxytocin is a key to subsequent physiological regulation that follows (Hofer, 2006). The perception of safety is vital to maintain oxytocin levels to reduce stress and is provided by immediate and continuous SSC. This supports regulation at birth—transition to extrauterine life—as described above (Bergman et al., 2004; Chi Luong et al., 2016).

Olfactory cues have been proposed as the primary reassurance for the newly born that it is “safe” (Varendi et al., 1998; Schaal et al., 2020). Smell of the colostrum triggers activation of the prefrontal cortex (Fulbright et al., 1998) via the olfactory bulb and amygdala (Bartocci et al., 2000), evidence of an oxytocin-based emotional and social approach (Schore, 2001a).

#### 2.6.2 Critical period

The separation paradigm dismissed the concept of the “early critical period” operating briefly at birth as described by Konrad Lorenz in imprinting in goslings (Moriceau and Sullivan, 2005; Mobbs and Mobbs, 2015). Assumptions and beliefs concerning the immaturity of the human brain allowed for later and “longer sensitive periods” in development and continued separation at birth were justified on this assumption. The iKMC results suggest that the early critical period is indeed operating, with mechanisms not yet fully known.

As much as all maternal sensory sensations provide regulators, no single factor will explain this. The entire oxytocin system “shapes environment-dependent neurobiological systems” and charts “the first integration of brain and environment in human life” (Carter et al., 2020). In terms of immediacy, even mode of delivery with concomitant separation (vaginal versus caesarean) provides altered signaling with epigenetic inputs evident over the life span (Kenkel, 2021). SSC has been shown to reduce neonatal oxidative stress in 3-day-old infants (Forde et al., 2020), and this effect operating immediately after birth may be critically important. Feldman suggests that maternal regulation through SSC may be setting infant biological clocks or oscillators, which is evident in cardiac vagal tone, cortisol reactivity, and organization of sleep and waking (Feldman et al., 2014). The microbiota is seeded during and immediately after birth (see further below). There may be an early impact on maternal neuroplasticity which is maximal at birth (Strathearn, 2011; Kim et al., 2016).

In all of the above, oxytocin plays a major role and likely through a unique pulsatile mode of release (Carter et al., 2020). This is particularly important for neural plasticity, by which it “coordinates birth according to favorable environmental conditions … (with) massive epigenetic inputs, particularly related to attachment experiences” (Carter et al., 2020).

#### 2.6.3 Prefeeding behavior at breast

In 1977, Michel Odent presented a study describing the “early expression of the rooting reflex” appearing in babies left undisturbed with their mothers after birth (Odent, 1977). Following on this study, in 1987, Widstrom et al. reported on a “sequence of prefeeding behavior” during the first hour of undisturbed newly-born infants, who “found the nipple and started to suckle” (Widstrom et al., 1987). This was disrupted by gastric suctioning, as it would obviously be by separation. Similar observations and disruptions by health care practices were described in similar terms by Righard and Alade (1990). In dissemination of this research, the term “self-attachment” arose and became common parlance; in a guest editorial by Righard, the term appears in an added note promoting a video entitled “Delivery Self Attachment” (Righard, 2008). Attachment was the term used by Bowlby for the mother-infant attachment, Widstrom and Righard used the term attachment as Bowlby and do not refer to self-attachment, even in later publications (Widstrom et al., 2010, 2019). In lay literature, the term “breast crawl” has become popular (UNICEF, and Prashant Gangal, 2007), crawling is however only one part of the prefeeding behavior.

The original term used by Widstrom best fits the underlying scientific rationale. Prefeeding behavior is fundamentally mammalian reproductive biology, with suckling of the newborn as the objective (Widstrom et al., 1987). In most instances, colostrum is present in the first hour, and the infant will swallow it. Colostrum has many benefits, including immune protection (Pletsch et al., 2013), with lactose acting as inducer of innate immunity (Cederlund et al., 2013). Colostrum is however of insufficient volume to be nutritive, and lactose is present but in half the concentration of that in mature milk (Saint et al., 1984). Colostrum is however not always present, and during the first 2 days, suckling is therefore not primarily nutritive. The suckling behavior is fundamentally mammalian and an important prelude to subsequent feeding of full volume breastmilk (Salariya et al., 1978; Thomson et al., 1979), extra contact, and suckling in the first hour increased breastfeeding duration (De Chateau and Wiberg, 1984). As an oxytocin-based behavior, suckling requires first the perception of safety and then immediate regulation in skin-to-skin contact (right place). Even so, the suckling as such stimulates the back of the palate to provide a parasympathetic stimulation that further supports regulation (Foster et al., 2016), as well as activating oxytocinergic system effects (Uvnas-Moberg, 1996). The original description should be preferred: “sequence of prefeeding behaviour leading to suckle” (Widstrom et al., 1987).

Alberts, in mammalian studies, describes suckling as congenital (present at birth) but not innate in so far, as the newborn needs familiar olfactory cues to express nipple seeking and suckling behavior, as a first part of learning and development toward breastfeeding (Alberts, 1994; Alberts and Ronca, 2012). The role of olfaction has long been recognized in human fetal and neonatal period “in the realms of self-regulation, emotional balance, feeding, and social interactions” (Schaal et al., 2004). At birth, breast odors elicit head orientation, nipple localization, and suckling (Porter and Winberg, 1999). Doucet studied 3-day-old human infants, demonstrating that smell of donated areolar gland secretions elicited “inspiratory activity and appetitive oral responses … independently from direct experience with the breast or milk” (Doucet et al., 2009).

Smell independently elicits responses in the olfactory bulb that connect to the amygdala (emotional brain) eliciting a social approach response (Bartocci et al., 2000), and these are part of the oxytocinergic system (Uvnas-Moberg, 1996). Schaal describes the role of smell as a scaffold for development over the life span (Schaal et al., 2020). Over and above prefeeding behavior, olfactory attraction to breast is matched by attraction to familiar maternal odors, which “regulate the child’s emotions, scaffold perception, and learning through non-olfactory senses,” even in adulthood. Conversely, the absence of maternal odor can disturb “adaptive reciprocity between offspring and carer during the multiple transitions of development” (Schaal et al., 2020).

Our current care paradigm encourages SSC only for the first hour (Vincent, 2011; UNICEF, 2015). The implication is however that SSC is needed as the right place for ongoing expression and development of this behavior. This is recognized in new recommendations on SSC after birth (Brimdyr et al., 2023). The separation paradigm thus interferes with prefeeding behavior in preterm and full-term newborns. The oxytocin paradigm regards the prefeeding behavior as normal and ordinary infant behavior.

#### 2.6.4 Suckling and preterm birth

Preterm separation currently remains standard in most parts of the world (Engmann et al., 2013; Raiskila et al., 2017). The entire oxytocin milieu and safe place needed in the first hour, in which regulation and prefeeding behavior occurs is absent. High levels of cortisol are maintained by higher level perception of threat, so oxytocin is suppressed. The prefeeding behavior and suckling cannot therefore be expressed. Feeding by bottle is directly dangerous in such a situation (Meier, 1988; Chen et al., 2000), and tube feeding is necessary. There is uncertainty whether these should be oral or nasal, “more evidence needed” (Watson and McGuire, 2013). Both are however acknowledged as stressful (Dsilna et al., 2008), which the separation paradigm accepts.

A report published in 1994 from the NICU in Uppsala, Sweden, noted the “importance of skin-to-skin contact during the mother’s first visit” (Nyqvist et al., 1994). In this context, the prefeeding behavior was observed, even in very preterm infants, and developed into a “Preterm Infant Breastfeeding Behavior Scale” (Nyqvist et al., 1999). Infants were enrolled at 28 weeks, at which time, they showed “efficient rooting, areolar grasp and latching,” and “nutritive sucking from 30.6 weeks.” “Very preterm infants have the capacity … sufficient for the establishment of full breastfeeding at a low postmenstrual age” (Nyqvist, 2008).

Small and sick babies should be cared for in SSC, but they should also have care that supports the innate capacity to develop and maintain suckling. The suckling is a highly conserved neuroendocrine behavior and is entirely dependent on the safe place. Experience and anecdotes of this are reported widely, but the separation paradigm still has difficulty implementing the first hour non-separation and suckling for full-term infants and still rejects it for preterm infants.

#### 2.6.5 Role of colostrum

In the final stage of labor, there is a peak of oxytocin which remains high through the first hour after vaginal birth (Uvnäs-Moberg et al., 2019). Likely attributable to this, it has recently been shown that there is a significant amount of colostrum in the breast at this time (Parker et al., 2012, 2015). The clinical application of this is that for preterm infants that are denied the opportunity of early suckling, it is vital to express colostrum from the mother during the first hour. Expression should be done in addition to supporting suckling for smaller and sicker babies. If first expression is delayed to the second or third hour, almost nothing will be obtained. Furthermore, the first hour expression and subsequent frequent expressions greatly increase subsequent milk volumes at 6 weeks and beyond (Parker et al., 2015).

We may have been unaware of it, but likely full-term infant self-attachment supported by the Baby Friendly Hospital Initiative has led to this normal biological benefit (Garofoli et al., 2023). However, the role of colostrum has been downplayed by our current separation paradigm, and it deserves greater attention, especially for the preterm neonate (Slouha et al., 2023).The first hour colostrum will almost certainly contain high levels of oxytocin, and this has an important role in multiple gastrointestinal functions that are subjected to neuronal regulation (Welch et al., 2014a). The enterocytes (stomach lining) have been shown to have oxytocin receptors, contributing to early protection against inflammation (Gross Margolis et al., 2017). At the time of weaning, these receptors are expressed deeper down (endoplasmic reticulum), where they continue the direct link to the vagal system for ongoing regulation (Klein et al., 2017). Furthermore, colostrum has been shown to come with healthy microbiota likely derived from the maternal gut (Du et al., 2022). The oxytocin in colostrum likely protects the neonate gut during microbiome colonization from contact with mother or from hospital environment in her absence (Dominguez-Bello et al., 2010, 2016). It likely continues a protective role until breastfeeding is established on the second or third day of life (Klein et al., 2017). Maternal factors in colostrum and early milk directly support the early establishment of a healthy microbiota (Sánchez-Salguero et al., 2021).

#### 2.6.6 Emotional connection

Welch presents the term “emotional connection” between infant and mother (Welch et al., 2014b; Hane et al., 2018), with a practical appraisal tool for measuring infant biobehavioral stress responses early in infancy (Frosch et al., 2019). Eye-to-eye contact is a powerful stimulus for oxytocin and emotional connection. In the prefeeding behavior described above, “looks at face” of mother by infant peak in incidence from 20 to 40 min after birth (Widstrom et al., 2010), the inference is that the emotional connection is ongoing in this place and early time.

Panksepp identifies the “integration of emotional systems for social affect” as foundational for healthy development, with place attachment mechanisms operating with thermoregulation at birth as critically important (Nelson and Panksepp, 1998; Panksepp, 1998), which is mediated by opioid, oxytocin, and norepinephrine systems. Schore describes in detail the neurological connection taking place in the neonate between the emotional brain (amygdala) and the social brain in the orbitofrontal cortex and other parts of the prefrontal cortex responsible for executive function (Schore, 2001a,b). In preterm infants, these connections have been shown with MRI (Als et al., 2004) and EEG (Welch et al., 2014b). A socioemotional approach response arising from a sense of safety follows (Minagawa-Kawai et al., 2009) facial recognition of mother and eye-to-eye contact. Face recognition takes place in the fusiform gyrus, which is directly linked to the amygdala (Petrovic et al., 2008). Porges details the close connection of the cranial nerves to the face with the autonomic nervous system (Porges, 2001) and the autonomic nervous system as a deep source of emotions (Porges, 1997). Meltzoff first described babies’ imitation of parents’ faces in the first day of life (Meltzoff, 1999), and frontoparietal mirror neurons accomplish this (Lenzi et al., 2009), with connections to both the amygdala and insula, another key center for emotion regulation (Ponserre et al., 2020). Lenzi suggests that empathy is thus generated by the inner imitation of actions of others. In the same way, Ross states “oxytocin increases gaze to the eye region of human faces and enhances interpersonal trust and the ability to infer the emotions of others from facial cues” (Ross and Young, 2009).

It deserves to be emphasized that the integrated “emotional systems for social affect” are the earliest and first neurobiological systems required for healthy development (Panksepp, 1998). Furthermore, the full sensory maternal input operates, with ongoing emphasis on olfactory, somatosensory, and auditory inputs providing reassurance of the “safe place.” One key component contributing to this emotional system is connections to the dopaminergic system (Feldman, 2020), though multiple systems must interact (Charney, 2004). Dopamine has widespread and vital neuromodulator effects on mood and behavior and, specifically, close connections to the oxytocinergic system mediating socioaffiliative behaviors (Baskerville and Douglas, 2010). Effectively, a powerful motivation and reward element are added to the emotional connection so that oxytocin-driven sociality becomes rewarding (dopamine) for both mother and baby.

#### 2.6.7 Maternal sensitization

The emotional connection is primarily to mother but does work for another caregiver. The role of oxytocin in the mother during pregnancy, labor, birth, breastfeeding, and caregiving is well established. A point of emphasis in this context is that oxytocin physiology is unifying, providing a single common thread for an overarching purpose over the whole period of reproduction (Feldman, 2020). It is also the enabler for reproductive ecology, in which organisms interact in the environment, and in this case, the environment for the newborn is the mother (Hrdy, 1999). The neural circuitry connections made in the baby allow for resonance and reciprocity within pre-existing identical circuitry in the mother (Swain et al., 2007). However, this circuitry is augmented in the mother by the combination of high oxytocin and her newborn’s sensory stimulations, specifically the suckling in the first hour, vocalization, and tactile stimulations (Strathearn, 2011; Velandia, 2012).

In humans, pregnancy has been identified as a sensitive period with opportunity for health-promoting influences and risks for mal-adaptation (Davis and Narayan, 2020). Mammalian studies show increased dendrification of parenting brain centers in late pregnancy (Kinsley and Lambert, 2008). This is the basis for neural plasticity, whereby sensory stimuli from offspring are needed to “fire and wire” circuits and networks made possible by pregnancy. Detailed studies on animals show species variability, with common threads related to multiple hormone systems. In some animals species (e.g., goats and sheep), offspring must work hard to overcome rejection/avoidance that is the normal baseline state in adults (Keverne and Kendrick, 1994), as if to prove that it is fit to invest in.

The human neonate that suckles is stimulating prolactin which is needed for alveolar development and lactogenesis (Trott et al., 2008). Oxytocin and cholecystokinin may induce an overall sense of warmth, wellbeing, and self-efficacy in the mother (Uvnas-Moberg et al., 1987; Weller and Feldman, 2003). Baby hand movements on the skin and areola may potentiate oxytocin to support this but also identify for the mother and baby as the object of attachment and for maternal ferocity in defense of young (Uvnas-Moberg and Francis, 2003). In all of the above, the newborn is wiring the maternal brain to ensure its basic biological needs: food (prolactin), warmth (cholecystokinin), and shelter (oxytocin). This neural plasticity is evident in MRI studies that show an increase in size of regions responsible for fear and reward processing, emotional regulation, and executive function with empathy (Kim et al., 2016).

In terms of direct clinical evidence, a careful study in Russia showed that early and direct SSC in the first 2 h after birth led to greater maternal sensitivity, infant self-regulation, dyadic mutuality, and reciprocity at 1 year as compared with those held in arms while clothed (Bystrova et al., 2009). The Immediate Parent Infant Skin-to-Skin Study (IPISTOSS) was formulated in parallel with the Immediate KMC Study (Adejuyigbe et al., 2020; Linnér et al., 2020). The actual method of SSC was developed in Karolinska, Sweden, and implemented identically in the iKMC study by the same team. The IPISTOSS skin-to-skin contact intervention lasted only for the first 6 h after birth, during which time controls were separated while receiving identical state-of-the-art intensive care. The parental regulation of the baby in SSC was demonstrated, which was reported in the primary outcome of improved transition to extrauterine life (Linnér et al., 2022; Lode-Kolz et al., 2023). Notably, in this context, at 4 months, there was higher quality of mother-infant interaction in the SSC group (Cohen d = 0.67 [95% CI, 0.17 to 1.17]; *p* = 0.01) (Lilliesköld et al., 2023). In another study, 1 h of SSC intervention in mothers and fathers increased oxytocin and reduced stress and anxiety in the first 2 days of life (Cong et al., 2015). Short SSC episodes increased oxytocin and decreased cortisol with left frontal brain activation (Hardin et al., 2020).

The above sampling of clinical evidence reports higher oxytocin and behavioral benefits from the intervention of SSC. However, the new paradigm and novel interpretation proposed is that SSC is not an “intervention.” First, it is the biological “normal” to which interventions (such as separation) should be compared. Second, it is a place for care and intervention rather than an intervention or care as such.

Optimal reproductive physiology is place- or habitat-dependent. Disturbing or altering the infant’s expectations in any way leads to disruptions, and maternal–infant separation is the intervention that leads to the most severe harm to newborns in non-human primates (Parker and Maestripieri, 2011). The separation paradigm does not allow or admit that care practices cause harm, only that doing something else may have benefit and is acceptable.

#### 2.6.8 Parenting brain

The oxytocinergic and dopaminergic systems are fundamental for maternal caregiving behavior and parenting (Strathearn, 2011). Several neurobiological connections between the two systems are described, and the importance of infant cues such as suckling, vocalization, and tactile stimulation in connecting them (Strathearn, 2011). This makes care for the newborn rewarding for the mother and also compulsive with an addictive quality (Swain et al., 2007). Dopamine supports habitual behavior, which may contribute to making this care easy and less stressful. In this context, oxytocin as the affiliation hormone is for parenting but now augmented by dopamine empowering it with purpose, reward, and habitual behavior (Swain et al., 2007). This is likely the underlying mechanism for documented short- and long-term psychophysiological benefits from mothering and parenting (Labbok, 1999; Buckley, 2015; Bartick et al., 2017).

Where such connections are absent or weaker, whether from maternal childhood adversity or current circumstances, maternal caregiving behavior is affected, and maternal neglect may follow (Strathearn, 2011). Activation of dopaminergic and oxytocinergic maternal brain regions by infant stimuli is measurable in MRI studies in mothers and fathers, with concomitant oxytocin changes in the blood stream. Mothers identified as “synchronous” in their interaction with their baby show this clearly, and it is absent in those identified as “intrusive” (Atzil et al., 2011). Vaginal birth supports oxytocin and dopamine connection, whereas elective caesarean birth does not support the same (Swain et al., 2008). An extensive review of such studies in parenting behavior uses the term “contingent interaction” in describing the resulting mother-infant behavior. The tighter this interaction, the better the infant’s development and long-term outcome; multiple disruptive factors are identified that incrementally weaken the interaction, including “maternal separation” (Swain et al., 2007).

#### 2.6.9 Breastfeeding and sleeping

The SSC on the chest of mother is the safe place for achieving newly born regulation, prefeeding behavior, emotional connection, and priming the maternal brain for parenting, and all have underlying neuroendocrine behaviors unique to this early initiation period. Suckling and ingestion of colostrum are distinct and can therefore be regarded as precursor achievements toward subsequent breastfeeding and ingestion of mother’s own milk, which will only begin on the second or third day. Therefore, in the new WHO guidelines, SSC should be initiated immediately and then be provided “8–24 h per day (as many hours as possible)” (WHO, 2022b). In the iKMC study, breastfeeding as an intervention was controlled, and both groups were given colostrum in the first hour and frequently thereafter. As stated earlier, there was no difference in breastfeeding rate at 28 days, but what is noteworthy is that the control group achieved an 85% rate of exclusive breastfeeding (86% in SSC) (Arya et al., 2021). This high rate reflects some resilience in the prefeeding and suckling behaviors (Nyqvist, 2008), and the early and frequent colostrum expression (Parker et al., 2015) as the primary contributor in ensuring milk supply improved exclusive breastfeeding.

Suckling has been described as a primary occupation of the newborn (Alberts, 1994), the second is emotional connection as described above (Hane et al., 2018), and the third is sleep (Bergman, 2019a). All three are oxytocin system supported and dependent on safe place as evidenced by research on SSC. Neonates do not sleep like adults, and there is an apparent care expectation that they should do so, with supportive advice for parents to sleep throughout the night. Adult sleep is determined by the circadian rhythm, in which light from the sun regulates a master clock or “zeitgeber” (von Schantz et al., 2000). This is understood to regulate all other molecular clocks and is termed chronomics (Papaioannou et al., 2014). Such clocks are numerous and found in almost all cells (Kwon et al., 2011). There is however “another somewhat mysterious oscillator, the food-entrainable oscillator” (Green et al., 2008). This is expressed by a clock gene identified in neurons of the dorsomedial hypothalamus. This connects, in turn, to the lateral hypothalamus, which controls regulation of the sleep/wakefulness and fasting/feeding cycles. Clocks in peripheral tissues such as the liver also can be entrained by food (Green et al., 2008). Therefore, the zeitgeber for the primary occupations of the neonate is feeding. Smell has been identified as the primary stimulus indicating safety, but it also regulates the coordinated feeding and sleeping cycle. The smell of areolar gland secretions specifically maintains sleep integrity and breastfeeding behavior (Doucet et al., 2009, 2012).

#### 2.6.10 Ultradian sleep rhythms in the perinatal period

The fetus shows an ultradian sleep rhythm with an evolving pattern through gestation (Koyanagi et al., 1993). At term, this shows a stable 1-h rhythm of alternating active and quiet sleep, where active sleep is prevailing and continues to be so in the first postnatal months (Peirano et al., 2003). Sleep cyclicity in preterm infants in SSC showed very similar 1 h of cyclicity, a mean of 68 min (Scher et al., 2005).

The orexinergic system has neurons in the posterior lateral hypothalamus that maintain wakefulness; sleep-promoting neurons are found in the ventrolateral preoptic nucleus (Saper, 2013). In rat studies, maternal deprivation increased cortisol and increased wakefulness at the expense of sleep through increasing expression of orexin receptors, leading to adult insomnia (Feng et al., 2007). In rhesus monkeys, maternal separation increases cortisol with subsequent disturbed sleeping (Barrett et al., 2009). Disturbed sleep “interferes with the normal restorative functions of NREM and REM sleep,” and REM is important for regulation of emotion (Brown et al., 2012) and for optimal physiological function and health (Bennet et al., 2018; Durankus et al., 2020). Infants born preterm do have increased disturbed sleep with a “predominance of attention problems, and negative emotionality is related to sleep disruption” (Caravale et al., 2017). The same was found in preterm-born preschool children, who also had increased gastroesophageal reflux (Durankus et al., 2020).

#### 2.6.11 Ultradian feeding rhythms in the perinatal period

The fetus swallows amniotic fluid, and this is emptied by gastric peristalsis. This can be observed in ultrasound; when examined in videotape, a 40-to-50-min rhythm of swallowing and peristalsis was observed (Sase et al., 2005a). This rhythm first appears at 13 weeks gestation and remains relatively constant to term (Goldstein et al., 1987; Sase et al., 2005b). This rhythm is not reported for neonates, since swallowing is not provided on an hourly basis. Nevertheless, gastric emptying time remains very much the same as in the fetus, which reported as 36 min (Ewer et al., 1994) and 48 min (Cavell, 1981), being twice as long with formula feeds. One report states that compared with formula fed, “a fasting state recurred more rapidly in breast-fed infants” (Tomomasa et al., 1987), and feed interval was 3 h. Human breastmilk has extreme low energy density compared with all other mammals, being “high in carbohydrates and low in fat and proteins, leading to shorter bouts of infant satiation and requiring frequent feeding” (Gettler and McKenna, 2010).

Signals from the olfactory bulb reach hypothalamic nuclei that control sleep and feeding, coordinating 1-h sleep cycles with suckling at the breast (Green et al., 2008). One hourly feeds translate to approximately 20 mL of feeds for a 3-kg infant, which is also the size of the neonatal stomach (Bergman, 2013) and also the volume of a single milk ejection reflex (Prime et al., 2007). Based on observed fetal stomach growth being linear, this translates to feeds of 7 mL per kg of infant weight (Bergman, 2013). Based on the ultradian feeding zeitgeber determining the rhythm, this feeding volume by weight remains constant in the first weeks of life. In total, 1 h is also the healthy sleep cycle, which is critical for healthy development (Graven, 2006; Peirano and Algarin, 2007).

Gastroesophageal reflux is a frequent infant disorder, and Douglas presents findings on this based on “research from the perspective of evolutionary biology” (Douglas, 2005). This perspective includes “unrestricted access to the breast” with frequent suckling day and night, concluding that reflux is a “misalignment of biology and culture.” Biology expects a 20 mL of feed, culture provides 60 or 80 mL, and reflux is therefore the result of volume overload (Bergman, 2013). Assumptions and beliefs based on separated infants have not allowed that the biological expectation can be observed. The oxytocin paradigm does provide a scientific rationale: the newly-born infant needs the safe place on mother for oxytocin to operate, with prefeeding behavior leading to frequent suckling and healthy sleep cycles.

#### 2.6.12 Circadian rhythms develop later

The fetal 1-h ultradian rhythm should be supported early on and allowed to continue “naturally” until the circadian rhythm begins to better suit infant feeding and sleeping behavior after some months. The circadian rhythm appears after some months, a study reported “discreet physiological functions” attributed to cortisol at 8 weeks, rhythmical circadian melatonin at 9 weeks, and after 11 weeks expression of the gene for circadian rhythm (Joseph et al., 2015). Spangler reports that circadian adrenocortical activity first appears at 3 months and matures at 7 months, with considerable variability (Spangler, 1991). However, even in adults, healthy sleep architecture retains the 1-h sleep cycle, and the circadian rhythm allows that several cycles block together at night (Born and Wagner, 2009). In the infant, two sleep cycles will start aggregating through the day and night, independent of the circadian rhythm, when regular and frequent food intake is the priority. The circadian platform allows for subsequent entrainment to block more sleep cycles at night, as in the adult (Born and Wagner, 2009). The neonate should not be expected to have a circadian rhythm: the primary need for ongoing nutrition and brain-wiring sleep is regulated by a zeitgeber that is set at approximately 1 h. The circadian rhythm begins after 3 months, and sleeping through the night follows some time later.

Research and clinical practice have been conducted on separated infants in the absence of the maternal regulators necessary for the physiology of neonatal sleep and feeding. Interpretations of such research and practice recommendations are based on the separation paradigm. Furthermore, the separation paradigm has an element of reductionism, thus observes feeding and sleeping as independent behaviors. Assuming that separation is normal, observations of state organization define only awake and sleep. The Anderson scale for state organization has 12 levels defined without any reference to feeding (Chang et al., 2002). In a study on infants weighing 1,500 g, the “heart rate, body temperature, and sleep are running in a seemingly uncorrelated pace,” and this is attributed to “that stage of development” (Koch et al., 2021): it could more likely be due to lack of regulation from separation. Separated infants do not show physiological hourly sleep cycles that support neurodevelopment but rather several levels of stress, all of which resemble sleep, in particular the dissociation state (Perry et al., 1995). Similarly, in the first weeks of life. “sleep–wake and food-intake behavior is characterized by different ultradian periodicities, ranging from 2 h to 8 h” with ultradian rhythms decreasing over time (Lohr and Siegmund, 1999); separation can be presumed.

#### 2.6.13 Suckling and emotional connection

When the baby wakes the oxytocin milieu is obviously related to suckling or breastfeeding but is equally important for emotional connection with mother, with eye-to-eye contact (Ross and Young, 2009). This is often maintained during suckling, and left laterality of maternal cradling has been identified as part of emotional connection (Sieratzki and Woll, 2002).

Tronick presented a “mutual regulation model,” stating that “the earliest foundation for infant resilience is maternal regulation” (Beeghly and Tronick, 2011). Feldman provides an updated description of the early regulation, resulting emotional connection, and another on the role of oxytocin in sensitive periods (Feldman, 2015, 2020). This is based on reciprocal and contingent interactions in the dyad, with ongoing communication as a platform for emotion regulation. This has also been called “biological attunement” (Di Lorenzo et al., 2022), and mother and her newborn being “mutual psychophysiological caregivers” (Anderson, 1977).

#### 2.6.14 Resilience and vulnerability

Resilience has been defined as the “dynamic capacity of an individual to maintain or regain mental health following exposure to stress or trauma” (Rutter, 1993). More recent terms used in reviews are “the capacity to recover from distress” (Di Lorenzo et al., 2022) and a “positive outcome despite adversity” (Feldman, 2020). Resilience should be recognized as the primary objective of development, as the “ultimate goal of human maturity” (Feldman, 2020).

The package of physiological regulation with feed and sleep cycling and a sense of safety in emotional connection and mother-infant interaction is based on mechanisms founded on oxytocin (Feldman, 2020), linking to other neurotransmitter systems. Charney describes nine such systems and mentions others that are involved in resilience and its opposite: vulnerability (Charney, 2004). Specifically, dopamine reward circuits may reinforce oxytocin circuits for social behavior, while over-responsive fear circuitry may overwhelm reward and sociality circuits (Charney, 2004). Fear circuitry is based on cortisol, as is pain circuitry.

The connection between oxytocin and dopamine circuits allows for the capacity to maintain healthy emotional functioning in the after-math of stressful experiences, and this is expressed very early. The paradigm presented here is that resilience requires maternal regulation, which must begin with SSC after birth and be provided continuously.

In summary, maternal regulation ensures a package of three neonatal occupations (Alberts, 1994), with a regular hourly rhythm of emotional connecting, feeding, and sleeping, all mediated by oxytocin (Moberg et al., 2020). This likely begins to operate in a critical period at birth and immediately thereafter. It involves also the mother’s brain directly and the subsequent contingent interaction between mother and baby (Swain et al., 2007). The emotional connection established allows for buffering protection against toxic stress and the establishment of resilience, the primary developmental outcome (Feldman, 2020).

### 2.7 An oxytocin-based model for nurture

All the functions and roles of oxytocin described above can be observed as different aspects of “nurture.” Nurture may appear as an ambiguous word and is surprisingly difficult to translate into other languages. Merriam Webster dictionary provides three meanings for “nurture”:

- early nurture as in upbringing, or
- something that nourishes like breastfeeding, or
- “nature versus nurture” where nature represents genetic determination and nurtures the differences of expression of behavior.

The latter meaning speaks to the genomic (nature) and epigenetic aspects of development (nurture), the nourishing speaks to brain and body development, and the first meaning the resulting behavior or reproductive fitness in the broadest environment of the world. This matches the psychobiological central dogma as described by Panksepp (1998). The broader genome contains the full potential of our species, the connectome is the sum of optimal neural circuitry achieved in early development and behavior “everything else” (Panksepp, 1998). The definitions span molecular and cellular aspects through the brain and organism to the social and moral (Shonkoff and Phillips, 2000).

While oxytocin does have a popular label as the “love hormone” (Odent, 2001) and many other epithets, I suggest that “nurture hormone” would be an apt summary of all its functions and broader role. The current separation paradigm should be shifted to an “oxytocin paradigm.” Oxytocin provides a common thread for the entire process of reproduction, health, and well-being as well as defense and survival in terms of resilience.

Given the above, *nurture* is an apt term, and nurture’s relevance for human health is such that it does deserve its own *science*. The term “nurturescience” would have been regarded as an oxymoron in the old separation paradigm. It has however the purpose of focusing on a new paradigm that has a scientific rationale, without the assumptions, beliefs, and research results of the separation paradigm. The term nurturescience has been presented in a publication that differentiates it from current assumptions and beliefs in the neuroscience environment where maternal–infant separation has been the norm (Bergman et al., 2019). A companion publication describes the separation paradigm as a source of toxic stress to the newborn (Bergman, 2019a).

The term and concept of nurturescience provides a model and a scientific rationale to guide a new way forward in the practical application of care (Bergman et al., 2019). Maternal–infant separation is the antithesis of nurturescience. To counter the present separation paradigm that accepts maternal–infant separation as normal, the term “zero separation” is intended as a single overriding communication objective for perinatal public health (Bergman, 2019a). There is a slight negativity in the “zero” communication objective, therefore nurturescience provides a more positive term. Both are however derived from a scientific rationale, as a single coin has two sides.

#### 2.7.1 Nurturescience model

**Figure 4:**
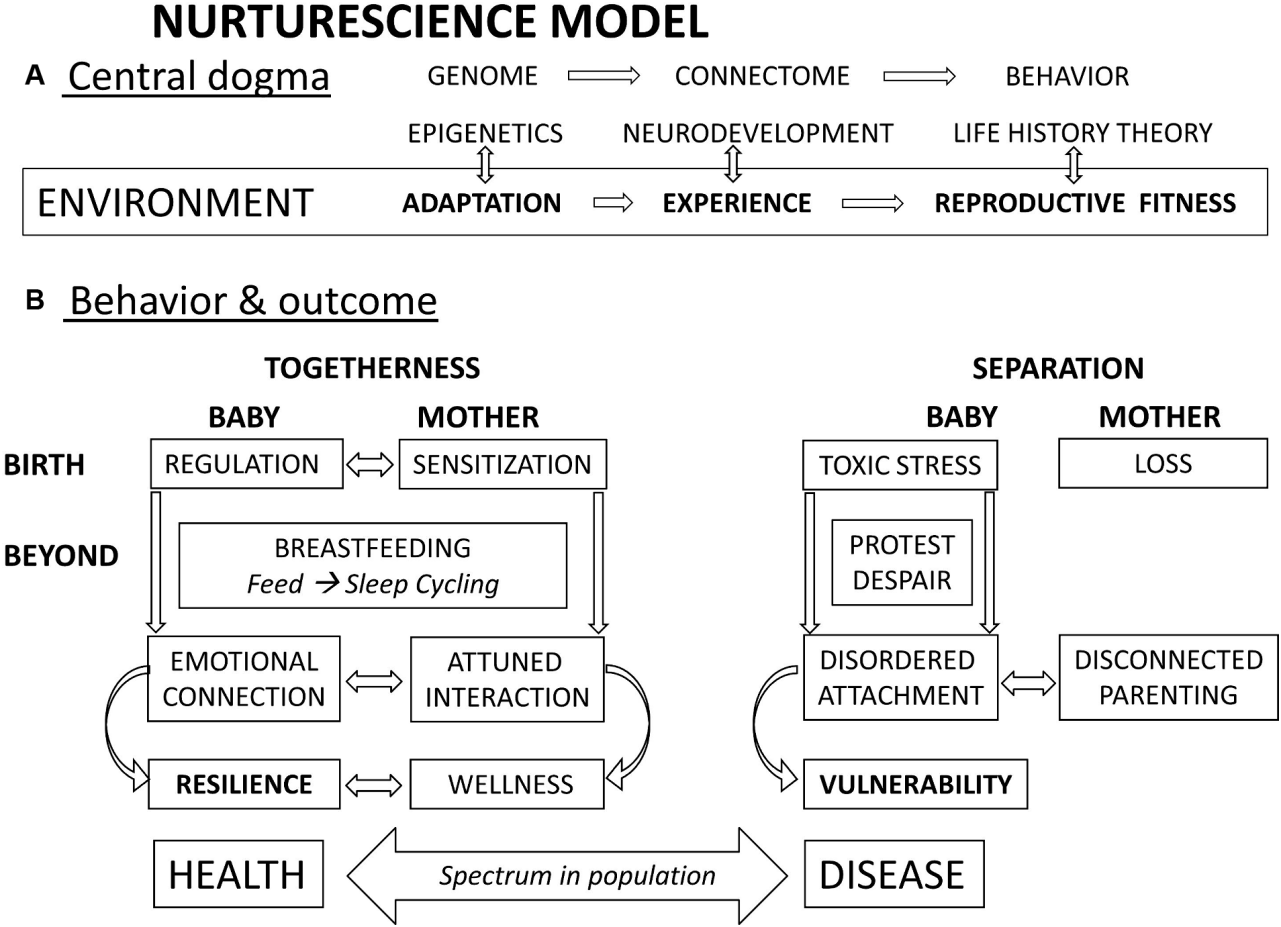
Nurturescience model. **(A)** The psychobiological central dogma described by Panksepp summarizes the scientific rationale for behavior and reproductive fitness. **(B)** Behavior at birth in immediate SSC ensures interaction between mother and baby achieving regulation of the baby and sensitization of the mother. “Beyond” this, continuous SSC is the place or environment in which salient sensory inputs lead to a global and dyadic neural interaction and behavior that includes feed and sleep cyclicity. Concomitantly, ensuring needs are continuously met, emotional connection in the dyad is matched by attuned or contingent interaction. This is the platform for lifelong resilience and wellness. In contrast, separation leads to poorer quality in all the above domains with no interaction, and a sense of loss for the mother. Toxic stress and the protest despair response in the infant may lead to disordered attachment in turn leading to vulnerability with risk for adverse impact over the lifespan.

The nurturescience model anchors its science in the central dogma described by Panksepp and the key role of the place or environment (Panksepp, 1998) and is depicted in the upper portion of Figure 4. Below this, the nurture aspects are presented as the biologically expected newborn and mother interaction starting immediately after birth and continuing uninterruptedly beyond that. The nurturescience model identifies mother as central to nurture and developing infant resilience, and this begins immediately after birth.

Resilience that supports optimal health over the lifespan is the primary objective of development in this model. Oxytocin plays a key role throughout the period of development. In contrast, separation leads to elevated cortisol and toxic stress, leading to adverse impacts on newborn and mother, with increased risk of infant vulnerability and disease. It is important however to note that life history theory makes it clear that mother was never intended to do all this nurturing alone. The broader scope of oxytocin includes pair bonding, specifically, and family and broader context as described in the evolved nest. The “zero separation” proposed applies to the mother and parents as much as to their newborn: the mother should never be alone. Some cultures value privacy, but our underlying biology does not work well on “aloneness,” since essential oxytocin is fueled by affiliation. Our biology cannot change, but our culture can.

#### 2.7.2 Life history theory predicts outcomes

Life history theory proposes that early stress and adverse circumstances prompt “costly but adaptive strategies that promote survival and reproduction” (Ellis and Del Giudice, 2019). Adaptation to adversity is the fast life history strategy and has needed short-term benefits but at the long-term cost of vulnerability. The slow life history strategy allows for resilience, with better health outcomes. Resilience and vulnerability develop on the identical neurological platform, programing or adapting to early signaling (Charney, 2004). Hormones are messengers carrying out the response of higher level assessment of safe place and relationships. The development of resilience can be observed as the default mode, and the intervention of separation produces vulnerability. Maternal–infant separation should therefore be avoided at all costs.

“Each system is perfectly designed to give you exactly what you are getting today” (Deming, 2024). Since the current perinatal care system is perfectly producing the product it does, the product is assumed to be perfect. The paradigm for care has produced the system, and paradigm is assumed and not questioned. The product of the system is therefore not questioned either. The primary quality indicator used in the past was improving survival (White, 2004), and this has undoubtedly improved. However, the quality of neurodevelopmental outcomes has not improved (Twilhaar et al., 2018; Pierrat et al., 2021; Louis et al., 2022). Since the medical profession’s trust in the system is embedded in the paradigm, such poor outcomes are not attributed to the system but rather to intrinsic immaturity or need for further technological advances. As presented above, the improved infant survival has come at the cost of shifting from a slow to a fast life history, with concomitant vulnerability and adverse health impact on the lifespan. For the health system to change, the paradigm must change. The practical expression of the oxytocin paradigm is immediate and continuous maternal–infant skin-to-skin contact. The outcomes of this and the broader implications of supporting oxytocin physiology beyond SSC are optimal growth and neurodevelopment, with lifelong resilience. The slow life history strategy has benefits for the individual and society as whole (Shonkoff and Phillips, 2000).

### 2.8 Ethical perspective and implications

Current perinatal care is often more focused on pathology than on physiology. The ethical axiom “first do no harm”—*primum non nocere*—is well known even to the public. Another term for this in non-maleficence, and this is deeply embedded in perinatal care. Evidence-based medicine is the guiding principle, and this is measured as risk reduction. Perinatal health systems identify all and any possible risks, and interventions are applied to reduce or mitigate them. Medical litigation acts as a watchdog to ensure compliance. In response, health system managers further prioritize this risk reduction by ensuring job descriptions that reduce risks at all costs.

There are other ethical axioms that should guide health care, one of which is beneficence (Varkey, 2021). It is not enough to avoid harm, and it is also necessary to actively do good. This entails health enhancement and provision of benefit, not merely risk reduction. In the context of pathology and disease, risk reduction is correctly applicable. However, pregnancy and birth and early infant development are not diseases, the entire perinatal period is essentially normal human physiology. Pathology can occur and must be managed, but all critical health-enhancing events and processes that belong to physiology supporting health must also be ensured. Currently, evidence-based medicine understood as risk reduction is applied on risk reduction principles. Where risk includes the “possibility of harm,” many physiologically based needs are ignored or glossed over. The ethic of non-maleficence is prioritized above that of beneficence.

Current perinatal systems assume therefore that by decreasing risks, outcomes will improve. This has been referred to as the pathogenetic approach (Alivia et al., 2011), whereby identification of symptoms and signs and establishing a diagnosis allow for treatment and restoration of health. The assumption is that health is merely the absence of disease. The paradigm of salutogenesis (Strümpfer, 1990) is in alignment with beneficence, recognizing that active health enhancement as provided by oxytocin physiology is necessary. For newborns, this must begin with restoring the right place on mother, mindfully and purposefully supporting the physiology of the dyad first and foremost, with due regard for risk and pathology.

The non-maleficence or pathogenetic approach can be observed as reductionist. Randomized controlled trials must ensure control of all variables and allow comparison of one variable against one other variable, with outcomes measured by risk reduction. Monotherapy is preferred, and the individual is the target. In contrast, the salutogenic model must ensure the big picture. Variables are not controlled in real life, and ecology involves organisms interacting with each other and with their environment in different times and for different reasons. Physiology addresses the entire organismal system in the environment. Rather than ensuring that “nothing is wrong” as in pathology, in physiology “everything is right” applies. Very specifically for the neonate and baby, this requires maternal regulation and is mediated by oxytocin.

A third ethical principle is justice, meaning that all individuals should be treated equally (Varkey, 2021). Autonomy is a fourth, meaning that each individual should be treated in the best possible way, with respect and dignity and privacy. In adults all four ethical principles carry equal weight and should be considered together in making difficult decisions. However, children do not have autonomy. The Convention of the Rights of the Child makes a different overriding principle for ethical decision making, namely “a child’s best interests are of paramount importance in every matter concerning the child” (UNHR, 2017). It further elaborates best interests as “highest net benefit among available options.” In this respect, beneficence as in benefit provision can be seen as more important for the child than risk reduction. This is well communicated in a “Position Paper on the Rights of Infants,” expanding on needs and therefore rights of newborns and infants (WAIMH, 2016), that is fully aligned with the oxytocin paradigm. An additional and helpful ethical axiom is prudence, “a smaller present good is not to be preferred to a greater future good” (de Lazari-Radek et al., 2014). Our current health system pays lip service to balancing benefit and risks for the good of mothers and babies, but the perinatal care system is in fact geared only to reducing risk.

### 2.9 Perspective on new policies

The review on SSC and neuroendocrine mechanisms concludes that these are “clinically important for better perinatal outcomes” (Moberg et al., 2020). This is powerfully borne out by the subsequent publication of the Immediate KMC study showing decreased mortality in small and sick babies, as described above (Arya et al., 2021). In the light of the nurturescience and oxytocin paradigm described above, and the new WHO guidelines, some selected perspectives on the iKMC study follow.

Effectively, the immediate SSC intervention required the introduction of a whole set of potential risks, forbidden in the current care paradigm. Nurturescience in practice does acknowledge risks and puts safeguards in place for them, but presents also a scientific rationale for the provision of benefit, as in resilience for the enhancement of health. The iKMC study ensured optimal risk reduction to both groups. The reported 25% reduction in mortality, was therefore a direct reflection of beneficence and salutogenesis. In the oxytocin paradigm where mother is the normal place for a baby, it is separation that increases mortality: if togetherness is seen as normal then mortality of the separated babies in the iKMC study represents a 31% increase in mortality (from 12.0 to 15.7%). A recent commentary identifies separation of maternal and newborn care in the USA as a “systemic threat to survival” (Anjur and Darmstadt, 2023).

An editorial accompanying the publication of the new WHO guidelines (WHO, 2023a), states “KMC is not a typical health intervention but an instinctual parental behavior that is the foundation of care for all preterm or LBW infants, nested within comprehensive small and/or sick newborn care (SSNC)” (Darmstadt et al., 2023).” The terms “foundation” and “nested” capture the right and safe place described above, identifying also the distinction between parental behavior and current evidence-based practice.

Nurturescience justifies a health professional dedicated to health enhancement. Staff with risk reduction responsibility will and must do all the risk reduction first, risk reduction must happen. However, health enhancement must also happen, and at the same critical time. As stated above: “the best interests of the child are paramount” (UNHR, 2017). Current job descriptions are entrenched in systems in which risk reduction is paramount. Health enhancement is practically, and affordably, achieved by a lower paid health care responsible exclusively for health enhancement as described by nurturescience. In the iKMC study, none of the tasks of the KMC supporters was medical in any way, or hardly even nursing in nature. Essentially the job description was ensuring that oxytocin physiology was maintained without interruption, and the intervention was entirely non-medical. Though lowly paid, even the highest paid health professionals should recognize this cadre’s role as paramount, and work within the oxytocin paradigm.

#### 2.9.1 Perspectives on SSC terminology and practice

In the light of the above, terminology and definitions can be reviewed. Skin-to-skin contact is the term first introduced and most consistently used (Sosa et al., 1976; De Chateau and Wiberg, 1977; Thomson et al., 1979). When reporting in English on the work of Rey & Martinez, Whitelaw also uses the term (Rey and Martinez, 1981; Whitelaw and Sleath, 1985). The term “skin-to-skin care” may sound more clinical than “Kangaroo Care,” neither convey the science presented above. As presented, SSC from a biological view is the immediate and continuous provision of the safe place. From the oxytocin paradigm, separation (with minutes as unit of measure) should be recorded with awareness for potential harm. Current measures are SSC initiation time (should be zero), daily dose and duration of days. In the context of prematurity methods and technologies used should be described, and which family member provides the SSC. This creates significant complexity for the reductionist evidence-based approach.

As described above, the term Kangaroo Mother Care, or KMC, was first coined to denote a composite strategy for preterm care (Cattaneo and Tamburlini, 1997; Cattaneo et al., 1998b). The Cochrane review on KMC is based on this definition (Conde-Agudelo and Díaz-Rossello, 2016). These authors report confusion with terminology, KMC is often used synonymously with SSC, making the review very difficult. They resolve the problem by stating the “major component of KMC is skin-to-skin contact (SSC),” and then they include all trials of KMC, “with all its components, irrespective of duration of intervention, breastfeeding patterns, and time to discharge from hospital.” It is clear therefore that this review is not about KMC as defined, but only about the first component, namely SSC. Subgroup analyses guiding policy relate only to aspects of SSC. In this review, all studies included only babies deemed stable as stipulated (WHO, 2003), except for one. That was a small study from Ethiopia that did include unstable babies, with SSC initiation at an average age of 10 h (Worku and Kassie, 2005). This one study lends more than half of the weighting to the conclusion on lowering of mortality, without it the effect on mortality is not statistically significant.

#### 2.9.2 Critique of WHO KMC policy

The WHO Global Position Paper provides a definition of KMC as a care strategy for preterm and low birth weight infants (WHO, 2023a). However, this is introduced with the statement that “KMC is different from the routine skin-to-skin contact recommended for all newborns” (WHO, 2023a). This author disputes this statement on two counts: the underlying scientific rationale and proposed oxytocin paradigm are the exact same for all newborns, regardless of weight or gestation. The method, practicalities and techniques do not differ. Second, the term “skin-to-skin contact” is not restricted for all (full-term) newborns, it is widely used for preterm and low birthweight infants (Forde et al., 2022; Kristoffersen et al., 2023), including when begun immediately and provided continuously (Linnér et al., 2022). In the Cochrane review it is identified as the primary component of KMC and called “skin-to-skin contact” (Conde-Agudelo and Díaz-Rossello, 2016).

The WHO then provides a four-point definition (WHO, 2023a), as follows:

“KMC refers to skin-to-skin contact that is:

- for preterm or LBW infants, both well and sick
- continuous and prolonged (at least 8 h per day)
- accompanied by support for exclusive breastfeeding or breast-milk feeding
- closely monitored if the baby is sent home in KMC.”

The first is new, and to the public or lay audience, the concept of the marsupial kangaroo method of care is appropriate to prematurity. It is also new in that small and sick babies are explicitly included, in contrast to the earlier implicit requirement of “clinical stability.” The second is not actually new, but explicitly supports continuous, while not mentioning the more important aspect of immediate. The third is unchanged from the original definition. The fourth has watered down the original definition requiring early discharge as a component. The original component of “early discharge” should be viewed as highly contextual (Chan et al., 2016). There is no doubt that the hospital environment has intrinsic dangers, stay should not be needlessly prolonged, if and when care can be safely provided at home.

The terminology confusion identified in the Cochrane review continues in the WHO Global Position Paper and Implementation Strategy: the term KMC is used when actually SSC is meant. Even in the above definition, a baby is “sent home in KMC,” this really must refer to SSC. The iKMC study controlled very carefully for breastfeeding support, with very high exclusive breastfeeding at one month in both groups (85 and 86%) (Arya et al., 2021). Discharge policies differed by sites, but were not different in the groups at each site. Thus, it is only SSC that can be initiated immediately, and it should be provided continuously, even after discharge.

#### 2.9.3 Oxytocin and nurturescience perspective for KMC policy

In nurturescience, there is no medical intervention called KMC or kangaroo position, there is a place of care called skin-to-skin contact. In this normal expected place normal physiological behaviors will be expressed, chief among which are suckling and breastfeeding. There is mutual regulation between mother and baby, and emotional connection leading to resilience. In small and sick babies the physiology may need extra technological support, and any pathology needs management and treatment without separation. In full-term healthy babies, suckling and breastfeeding are physiological behaviors (outcomes) of being in the right place, and not an intervention; in the context of small and sick newborns “support” for breastfeeding does qualify as an intervention. Critically important for the small and sick however is the provision of physiological support and treatment of pathology while receiving immediate and continuous SSC, this is not mentioned in the WHO definition.

This author presents the following as a definition of Kangaroo Mother Care.

- skin-to-skin contact (SSC), initiated as early as possible and provided continuously
- support for nutrition based on mother’s milk, aiming for exclusive breastfeeding
- provision of the full package of small and sick newborn care, including safe discharge.

Place comes first (SSC), physiology second (breastfeeding), and pathology third (care). SSC is the place that ensures healthy physiology, it is paramount and precedes the package of care. SSC is the place where the infant feels safe and provides the ecological salience for development, releasing oxytocin from higher brain centers. In the oxytocin paradigm, place is the primary component of KMC, rather than a first intervention as such. Physiologically expected behaviors—suckling and breastfeeding—are supported. Interventions for care and treatment are provided in non-separation; in the iKMC study they were identical in both groups (Adejuyigbe et al., 2020; Arya et al., 2021). Separation exacerbates the physiological and pathological status of the child, leading to the need for even more intensive care.

SSC duration should continue until the baby indicates that it no longer needs it, and this is clearly communicated by the baby at a variable and unpredictable time (WHO, 2003). The oxytocin milieu encompasses maternal–infant interaction for ongoing upstream and downstream regulation. After SSC has consolidated emotional security, eye-to-eye contact is prioritized for social development, provided while on mother’s body but in carry care (Lozoff and Brittenham 1979). An objective criterion is weighing more than 1800 g, preferably 2,200 g, on thermal control grounds. The infant’s high surface to body weight ratio and proportionately higher metabolic rate means the infant cannot generate enough warmth on its own (Sinclair, 1971). This is a best practice statement, in the absence of evidence. It could anyway be ignored, SSC should continue until the baby has had enough (WHO, 2003).

The entire physiology of breastfeeding is determined by oxytocin, however deeper insight and application is now needed to apply this health-enhancing physiology to the unexpectedness of prematurity. Early and frequent expression of colostrum from the mother will ensure current nutritional needs and adequate future milk supply. The baby needs support for innate suckling behaviors, as well as breastmilk feeding in the interim, leading to full exclusive breastfeeding. The risk reduction mindset has refused volumes of colostrum and mother’s own milk “as the baby cannot swallow safely”: this is a myth, albeit correct in the separation paradigm. Even very preterm infants can safely swallow when in continuous SSC and given insightful support (WHO, 2022b), with cognizance of sleep–wake cyclicity (Ludington-Hoe et al., 2006a).

The immaturity in preterm infants requires that technological support in proportion to the degree of prematurity is provided. The oxytocin paradigm however requires that the place-dependent physiology continues (i.e., in SSC), and that the necessary technology is added. Being small does however not equate to being sick, there may be purely maternal reasons for shortened pregnancy (e.g., incompetent cervix). There may however be purely fetal reasons (e.g., chorioamnionitis), and also contributions from both sides (e.g., placental insufficiency). The provision of the full package of small and sick newborn care therefore applies (WHO, 2022b). In the provision of care to small and sick newborns, providing physiological care in the right place of SSC does require new training and skills, likewise for supporting early nutrition and breastfeeding, and even more so for managing pathological problems. This may seem counterintuitive and frightening for those dealing with extreme prematurity. The concomitant risks of care are increased, this is acknowledged, and was addressed at length in each of the iKMC study sites. The evidence is however that the smaller and sicker the baby is, the greater the need for the health enhancement of immediate and continuous SSC. With this comes also a greater need for a new skill set informed by a new paradigm and understanding of the physiology of oxytocin. At this stage the direct and exact evidence for the mechanisms may not be known. The oxytocin paradigm is however that of a total package of the physiological expectations of the baby and interactive maternal–infant regulation, not a reductionist “this or that.” To repeat, the iKMC study lowered mortality by “increasing risk” as perceived by the separation paradigm, but immediate SSC was effective because it greatly increased benefit, as in resilience.

#### 2.9.4 Skin-to-skin contact for fullterm and preterm infants

As described in the introduction, in the USA the term kangaroo care is used, where skin-to-skin contact is provided briefly to stable preterms (Stanford, 2024). The term SSC is reserved for full-term infants in the first hour, as in the systematic review reporting evidence that SSC increases breastfeeding (Moore et al., 2016). Full term infants are therefore allowed this immediate SSC for one hour, after which they are “separated as normal” according to the current paradigm.

The scientific rationale for nurturescience and the oxytocin paradigm is actually derived for full term births. The concept of SSC may have been first described in the literature for use in low birthweight neonates, but it is efficacious because the underlying biology is that of the human species as a whole. There is no evidence at all that full term babies benefit from such separation, whether before or after the first hour. Full term babies should also have immediate and continuous SSC. They may not need it for very long, and they will communicate their desire to transition to carry-care, where zero separation should continue but within an expanded social habitat.

Referring to the new WHO definition of KMC described above, this author stated that SSC for preterm and full-term neonates are the same. One acknowledged difference in practice is that what is recommended for all newborns is seen as applicable for the first hour only, unlike preterm and low birthweight infants. The problem is not with the contention for full-term infants’ need for continuous SSC, but the separation paradigm that assumes full-terms should be separated.

In summary, SSC is a fundamental part of the biology of all newborns, not only those born small and sick. Nurturescience, zero separation and the oxytocin paradigm apply even more to preterm and low birthweight infants. The more immature they are, the greater their need for maternal regulation, and the greater the risk of harm from separation. They will also need technological support for immature lungs and other organs, and this should be guided by the understanding of oxytocin physiology. Preterms are small but may also be sick, the new paradigm needs to add management and therapy for any specific pathology, while prioritizing and protecting the oxytocin physiology. Protecting the physiology will better support treatment provided, and also contribute to prevention of further pathology.

## 3 Actionable recommendations

### 3.1 WHO guidelines

The WHO guidelines should be implemented worldwide, in all contexts. The Global Position paper articulates this to international and national policy formulation, requiring leadership and allocation of resources (WHO, 2023a). The Implementation Strategy document likewise, but has more practical directives to health services and facilities (WHO, 2023b). A high quality evidence base is presented with strong recommendations from rigorous review (WHO, 2022b). A practice guide is under preparation.

The guidelines are actionable. They have a scientific rationale as elaborated in this article. The guidelines have an ethical imperative (WAIMH, 2016; UNHR, 2017). The critique presented here is partly based on semantics, and partly based on a perspective and deeper understanding of the scientific rationale. This does not change any of the recommendations but is rather intended to strengthen the case for the implementation of the guidelines.

### 3.2 Perinatal care systems change for immediate skin-to-skin contact

All newborns should start immediate SSC without separation. Physiological cord clamping facilitates this (more below). They should continue in SSC for the first hours or days. The birth of preterm or low birth weight newborns should be no different, their physiology and need for oxytocin is exactly the same. However, the harmful effects of oxytocin deprivation are much greater. The Immediate KMC Study intervention describes the technique and practices (Adejuyigbe et al., 2020; Arya et al., 2021), a training film (Bergman, 2017) and necessary infrastructure changes (Chellani et al., 2022). An important actionable recommendation is to ensure close collaboration and clinical cooperation between obstetrics and neonatology (Anjur and Darmstadt, 2023). Fetal care is not only the responsibility of the obstetrician, and hands-on neonatal intensive care should begin in the labor ward in SSC with the mother. Neonatal care on mother should continue wherever else her medical condition requires, until both are in the Mother-Newborn Care Unit (Chellani et al., 2022; Klemming et al., 2023). Obstetric and midwifery care should continue in the neonatal unit. Any change of room can be stressful, transporting should be done in SSC for buffering the infant from that stress, and surrogates are helpful for this to ensure zero separation. This is detailed in the WHO document for implementation of KMC (WHO, 2023b), which describes a broad systems approach to change, encompassing all six health system building blocks (Bergman et al., 2023).

### 3.3 Gentle birth and physiological cord clamping

There exists a considerable body of evidence on birthing practices that align with the oxytocin paradigm, detailing this is beyond the scope of this article. Nevertheless, the separation paradigm has entrenched a host of “common care practices during labor, birth … (that) have not proven efficacious” (Mercer et al., 2007). In terms of a new perspective, such practices increase neonatal cortisol and contribute to harm. Over and above components already described above in nurturescience, cord clamping should be “slow” and not “fast”: physiological cord clamping applies even more to small and sick babies (Andersson and Mercer, 2021; Seidler et al., 2023). The term “delayed cord clamping” implies that the cord is normally cut immediately, in the oxytocin paradigm the normal should change. Mercer summarized many years ago “recommendations support a gentle, physiologic birth and family-centered care of the newborn” (Mercer et al., 2007).

### 3.4 Family involvement

In the WHO guide to preterm care, there is a whole new section with four new recommendations on family involvement (WHO, 2022b), this should be “actioned” from the outset. As described above, to achieve continuous SSC and zero separation the mother needs family support. But the oxytocin paradigm underlies family involvement beginning long before birth, and particularly during labor and through the birthing process. During labor that family role may have an “asocial” dimension, for the mother to focus inward she needs to feel protected from external interference, while supported internally. This role can well be done by a doula or family member birth companion who can help the family as a whole in their new roles, and there is evidence for benefit for this (Kozhimannil et al., 2013; Sobczak et al., 2023). In the postpartum period the mother-infant emotional connection should be protected and supported, with gradual widening of direct family support.

### 3.5 Salient sensory inputs for perception of safety

Salient sensory inputs for critical periods for the newborn all come from mother. Extraneous sensory inputs require threat appraisal by the infant, and have potential to increase cortisol and lower oxytocin and disrupt “needed neural processes” (Graven, 2004). In a neonatal unit such inputs become tolerable when buffered in SSC (Sanders and Hall, 2018), but a stress environment can overwhelm mother and infant. This has long been recognized, though early pioneers met resistance for their ideas. NIDCAP describes such an environment (Westrup et al., 1997; Als, 2015), numerous Infant and Family Centered Developmental Care (IFCDC) programs likewise (Klemming et al., 2021). The systemically entrenched paradigm of care regards such an environment as ideal or utopian. It is seen as “nice,” but a luxury that only the rich can afford, given workload pressure. In terms of actionable recommendations, the oxytocin paradigm providing a new “constellation of beliefs, values and techniques” (Kuhn, 2008), with respect to the environment is necessary. The developmentally supportive sensory environment should be implemented in all delivery wards and neonatal units.

## 4 Discussion

### 4.1 Pace of care from slow life history strategy

The oxytocin paradigm fosters the slow life history with optimal development and resilience, rather than the fast life history with allostatic load and vulnerability. The “slow and fast” applies also to the new and old paradigms identified. The separation paradigm focused on pathology is fast and quick, perhaps inspired by the emergency room ethos, and work pressure. Labor is regarded negatively as painfully slow, the caesarean is quick and easy. Resuscitation should be rapid, the latch to the breast assisted. Facilities are understaffed, work efficiency is essential.

The oxytocin paradigm however requires a slower pace, adjusted to the maternal and neonatal physiological and metabolic processes, and the intrinsic competencies and behaviors of the dyad. Physiological processes are much slower, both at the organismal behavioral level and more so at the organ metabolism level. The oxytocin paradigm sees perinatal pathophysiology as a physiology that is overwhelmed rather than deranged (Stöppler, 2024), and channeled from wellbeing to survival (Charney, 2004). Observations and interventions are necessary but the manner and the timing of both should foster physiological recovery from pathology. Carter suggests that oxytocin is nature’s medicine, supporting “growth, resilience and healing … stress coping … influences the autonomic nervous system and the immune system” (Carter et al., 2020). This requires the safe place, foundation for an ongoing sense of perceived safety for the dyad, not to be confused with a clinical assessment of safety.

### 4.2 Measuring resilience and vulnerability

New concepts and perspectives allow for new questions and ideas. Nurturescience highlights the need for supporting resilience and preventing vulnerability in early development. The above proffered description of resilience – the capacity to maintain healthy emotional functioning in the after-math of stressful experiences – is practical in so far as it is measurable. A primate study reports cortisol measures before, during and after a stressful event, in which resilient mother-reared monkeys have cortisol levels that return to baseline within 15 min, the vulnerable peer-reared ones maintain high levels (Feng et al., 2011). A similar model would be provided by the still face paradigm with cortisol measurements. Vagal tone derived from heart rate variability has been used as such a measure (Feldman et al., 2014). There are likely many other ways to measure resilience. Resilience should be the primary objective of development (Feldman, 2020), it should have objective outcome measures.

Resilience comes from the health-promoting oxytocin paradigm, vulnerability comes from the separation paradigm. Vulnerability should therefore be recognized as a disease. It has few or subtle symptoms, but it has clear signs (even if derived by negation in measures of resilience), and could be confirmed by diagnostic tests. The oxytocin paradigm recognizes vulnerability as an adverse outcome, that requires identification, as in diagnosis. The utility of this concept is that it would create leverage for change to current perinatal care practices. If vulnerability is diagnosed early, the downstream diseases that are predicted in Developmental Origins of Health and Disease (Hochberg et al., 2011) may be averted.

### 4.3 Separation tolerance

In the separation paradigm, the concept of “separation tolerance” is an oxymoron, since separation is seen as the normal, and SSC is perceived as something the newborn may not tolerate and is deemed dangerous. Separation and social isolation in newborn non-human primates are too devastating to be permitted, research in monkeys is done on peer-reared compared to mother-reared (Feng et al., 2011; Baker et al., 2017). In the rodent model used for testing human antidepressant drugs mentioned above, the separation dose required was 3 min twice a day for 3 days (Ziabreva et al., 2003). In piglets 2 h a day from day 3 to day 11 of age was required to induce an anxiety and depression model (Kanitz et al., 2004).

An objective measure of separation tolerance could be useful. Toxic stress is defined as the “absence of buffering protection of adult support” (Shonkoff and Garner, 2012). Such buffering protection is totally provided by SSC. There are however some circumstances where SSC is not possible. Buffering is not “present or absent,” some protective buffering of adult support may be perceived by the infant from smell alone (Welch et al., 2014b), or from mother’s voice and touch. The *Octodon degus* depression model described above was prevented purely by maternal vocalizations during separation (Ziabreva et al., 2003). Separation tolerance may also be influenced negatively or positively by many other factors, e.g., gene allele variations, underlying personality, or influences during gestation. This complexity does not mean that the separation tolerance cannot be measured, only that research should address and tease out such issues.

Nobody has asked, much less studied, “what is the separation tolerance of *Homo sapiens*?.” In the IPISTOSS study, a 6 h non-separation period is reported as “improved” regulation in that time, and improved mother-infant interaction at 4 months (Lilliesköld et al., 2023). The new paradigm presents this as disrupted regulation at birth, with adverse impact on mother-infant interaction, this may contribute to infant vulnerability. Separation tolerance may be low in human babies.

### 4.4 Restoring the original oxytocin paradigm

A new paradigm with novel perspectives is presented, however a more accurate perspective is that this is more a case for restoring the original paradigm. “There is nothing new under the sun” wrote King Solomon (Bible, Ecclesiastes 1v10). More recently, Florence Nightingale (1820–1920) wrote (CurrentNursing, 2020): “Patients are to be put in the best condition for nature to act on them, it is the responsibility of nurses to reduce noise, to relieve patients’ anxieties, and to help them sleep.”

In conclusion, the WHO guidelines on KMC should be adopted and implemented worldwide: a better understanding of the science of oxytocin and nurture will facilitate this. This science is guided by oxytocin, the ancient hormone central to reproduction and growth, resilience and healing, sociality and love. The oxytocin paradigm as described in nurturescience restores dimensions missing in current perinatal care, including a deeper understanding of infant physiology, the ethics of beneficence, and the paramount “best interests of the child.”

## Author contributions

NB: Writing – review & editing, Writing – original draft, Software, Investigation, Conceptualization.
